# Cleavage and Polyadenylation Specificity Factor Subunit 5 Regulates Pulmonary Artery Smooth Muscle Expansion and Hypoxic Response

**DOI:** 10.1002/mco2.70610

**Published:** 2026-02-03

**Authors:** Scott D. Collum, Lisha Zhu, Tingting W. Mills, Rene Girard, Jamie Tran, Tinne C. J. Mertens, Cory Wilson, Nancy Wareing, Erik E. Suarez, Howard J. Huang, Rahat Hussain, Bindu Akkanti, Wenjin J. Zheng, Hari K. Yalamanchili, Bela Patel, Eric J. Wagner, Sandeep Agarwal, Harry Karmouty‐Quintana

**Affiliations:** ^1^ Department of Biochemistry and Molecular Biology, McGovern Medical School University of Texas Health Science Center Houston Houston Texas USA; ^2^ Data Science and Informatics Core For Cancer Research, McWilliams School of Biomedical Informatics University of Texas Health Science Center Houston Houston Texas USA; ^3^ Department of Internal Medicine Emory University School of Medicine Atlanta Georgia USA; ^4^ DeBakey Heart and Vascular Center Houston Methodist Hospital Houston Texas USA; ^5^ Department of Internal Medicine, Division of Pulmonary, Critical Care and Sleep Medicine, McGovern Medical School University of Texas Health Science Center Houston Houston Texas USA; ^6^ Department of Department of Pediatrics Baylor College of Medicine Houston Texas USA; ^7^ Department of Biochemistry and Biophysics The University of Rochester Medical Center Rochester New York USA; ^8^ Department of Medicine, Section of Immunology, Allergy and Rheumatology Baylor College of Medicine Houston Texas USA

**Keywords:** pulmonary arterial hypertension, RUNX1, cell proliferation, prostaglandin E receptor 3 (PTGER3), EP3, vascular tone, systemic sclerosis, CPSF5, SSc‐ILD

## Abstract

Pulmonary hypertension (PH) is a fatal condition that affects individuals with systemic sclerosis (SSc), a multiorgan fibrotic disease with limited treatment options. A central feature of PH is vascular remodeling, defined by the narrowing of the arteriole lumen due to cell proliferation and extracellular matrix deposition. Herein, we identify a central mechanism that can regulate multiple transcripts important for vascular remodeling. The highlight of our study is the demonstration that reduced pulmonary artery smooth muscle (PASMC) *Nudt21*, which codes for the RNA binding protein Cleavage and Polyadenylation Specificity Factor Subunit 5 (CPSF5) The, known to regulate alternative polyadenylation, results in heightened right ventricle systolic pressures in mice exposed to hypoxia–sugen. We also report that increased PASMC proliferation is present in mice with reduced PASMC *Nudt21* under normoxic conditions, recapitulating features of hypoxia–sugen exposure. Our studies reveal that reduced CPSF5 leads to 3′ untranslated region shortening of *PTGER3* and *CBFB*, the latter contributing to increased levels of proliferative transcription factor RUNX1. We also identify miR‐3163 as novel negative regulator of *NUDT21* expression in PH. These observations are validated in remodeled vessels from patients with SSc associated with PH and in and point to common mechanisms of RNA processing deficits that contribute to vascular remodeling in PH.

## Graphical abstract

Increased miR‐3163 targets *NUDT21*, reducing CPSF5 levels and driving APA dysregulation. This results in 3′UTR shortening of *CBFB*, which stabilizes RUNX1 and promotes smooth muscle proliferation. *PTGER3*, is also shortened amplifying hypoxia‐induced RVSP. Together, these pathways contribute to vascular remodeling in PH.

Image Created in https://BioRender.com

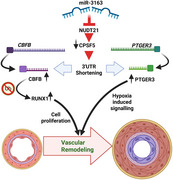

## 1 Introduction

Pulmonary hypertension (PH) is characterized by increased mean pulmonary arterial pressure (mPAP ≥20 mmHg [1]) and classified by the World Health Organization as Group 1 if it only affects the lung vasculature or Group 3 if it is associated with remodeling of the lung parenchyma [2, 3, 4]. A particularly understudied phenomenon is PH in the setting of systemic sclerosis (SSc). SSc (scleroderma) is a rare autoimmune disease that has the highest mortality among all connective tissue diseases [5]. SSc is a multiorgan connective tissue disorder characterized by immune dysregulation, vasculopathy, and excessive extracellular matrix (ECM) deposition, leading to skin and internal organ fibrosis [6, 7, 8]. The development of severe pulmonary complications such as interstitial lung disease (ILD) and pulmonary arterial hypertension (PAH) are the leading causes of death in patients with SSc [9, 10, 11, 12]. These disorders can present on their own or in combination with each other [9, 10]. In all of these cases, the diagnosis of PH is strongly associated with increased morbidity and mortality [2, 3, 13] and in the vast majority of cases, PH is not curable [14, 15]. Understanding the mechanisms that lead to the development of PH is essential for the discovery of novel therapies to treat this fatal condition.

One of the most striking observations of PH in all groups, including SSc patients, is the formation of complex vascular lesions in the lungs that increase blood flow resistance and increase mPAP. This process, known as vascular remodeling, is defined by the expansion of pulmonary artery (PA) endothelial and smooth muscle cells (PASMC) [1]. The mechanisms that promote the expansion of PASMCs are multiple and include increased expression of inflammatory mediators such as platelet‐derived growth factor (PDGF) [16], Hippo signaling [17], secreted protein acidic and rich in cysteine [18], prostaglandin E3 receptor [19] and altered expression of transcription factors associated with cell proliferation such as forkhead box O^20^, runt‐related transcription factor 1 (RUNX1) [21, 22], and others [1, 23]. A central inciting mechanism in the pathophysiology of PH is hypoxia (HX) [24]. Herein, several studies have demonstrated the capacity of stabilization of HX‐inducible factor in the expansion of PASMCs in both Group 1 and Group 3 PH settings [25, 26, 27, 28, 29, 30]. In addition to smooth muscle cell (SMC) proliferation and the role of HX in modulating these changes, PASMC phenotypic diversity has been identified to play a role in PH [31]. Despite identifying these mediators and processes, whether a unifying mechanism could explain the altered expression in vascular remodeling is unclear.

Provocative studies on epigenetics mechanisms and RNA biology have demonstrated how methylation and expression levels of bone morphogenetic protein receptor 2 are essential in regulating PASMC in PH [32]. Similarly, studies have shown that the epigenetic reader bromodomain‐containing protein 4 sustains PASMC survival and proliferation [33]. N1‐methyladenosine modification by the enzyme ADAR adenosine deaminase RNA specific (ADAR1) has been implicated in promoting PASMC proliferation in PAH [34]. These observations point to epigenetic processes in the pathophysiology of PH. In line with studies focusing on RNA processing, we have demonstrated a reduction of cleavage and polyadenylation specificity factor subunit 5 (CPSF5) and its gene Nudix hydrolase 21 (*NUDT21*) in remodeled vessels in patients with PAH [35]. These studies demonstrated how loss of *NUDT21* resulted in alternative polyadenylation (APA) and 3′ untranslated region (3′UTR) shortening of hyaluronan synthase 2 (*HAS2*), a gene coding for a key enzyme in the synthesis of hyaluronan, an ECM constituent associated with vascular remodeling in PH [36, 37, 38, 39]. These results indicate alterations in the cleavage and polyadenylation machinery as a potential upstream mechanism regulating PASMC proliferation and ECM deposition in PH.

Cleavage and polyadenylation (poly(A)) is required for stabilizing and translating mature mRNAs. Because most mammalian genes contain multiple poly(A) sites, transcripts can differ in their 3′UTR length through APA [40, 41]. Normally, the distal site is favored [42], but proliferating or differentiating cells often switch toward proximal sites, producing shortened 3′UTRs [41, 43]. This reduces regulatory elements such as microRNA (miR) and AU‐rich element binding sites, enabling increased protein expression. A key regulator of APA is cleavage factor Im 25 (CFIm25) also known as CPSF5, encoded by *NUDT21*, whose depletion drives global 3′UTR shortening [43, 44] and has been associated with enhanced oncogene expression and cell proliferation [43]. In pulmonary arterial hypertension (PAH), reduced CPSF5 expression has been reported in remodeled vessels and linked to 3′UTR shortening of HAS2, with consequent enzyme upregulation and increased hyaluronan deposition [35]. However, the broader contribution of *NUDT21*‐dependent APA to PH pathogenesis remains poorly defined.

In this study, we demonstrate that reduced PASMC CPSF5 in mice exacerbates hypoxia–sugen (SU) (HX–SU)‐induced PH and promotes vascular remodeling through two complementary mechanisms: direct upregulation of prostaglandin E receptor 3 (PTGER3) and indirect upregulation of RUNX1 through 3′UTR shortening and increased protein expression of core‐binding factor beta (CBFB). Together, this defines a novel upstream pathway leading to elevated PTGER3 and RUNX1, both of which have been implicated in the pathogenesis of PH [19, 21, 45]. Consistent with these findings, we show reduced expression of CPSF5/*NUDT21* in distinct presentations of PH, including SSc‐associated PH and PAH, accompanied by increased expression of PTGER3, RUNX1, and CBFB. These results highlight a common mechanism across different PH subtypes that contributes to vascular remodeling.

## 2 Results

### 2.1 Impact of SMC NUDT21 Reduction on HX–SU‐Induced Pulmonary Pressures

**FIGURE 1:**
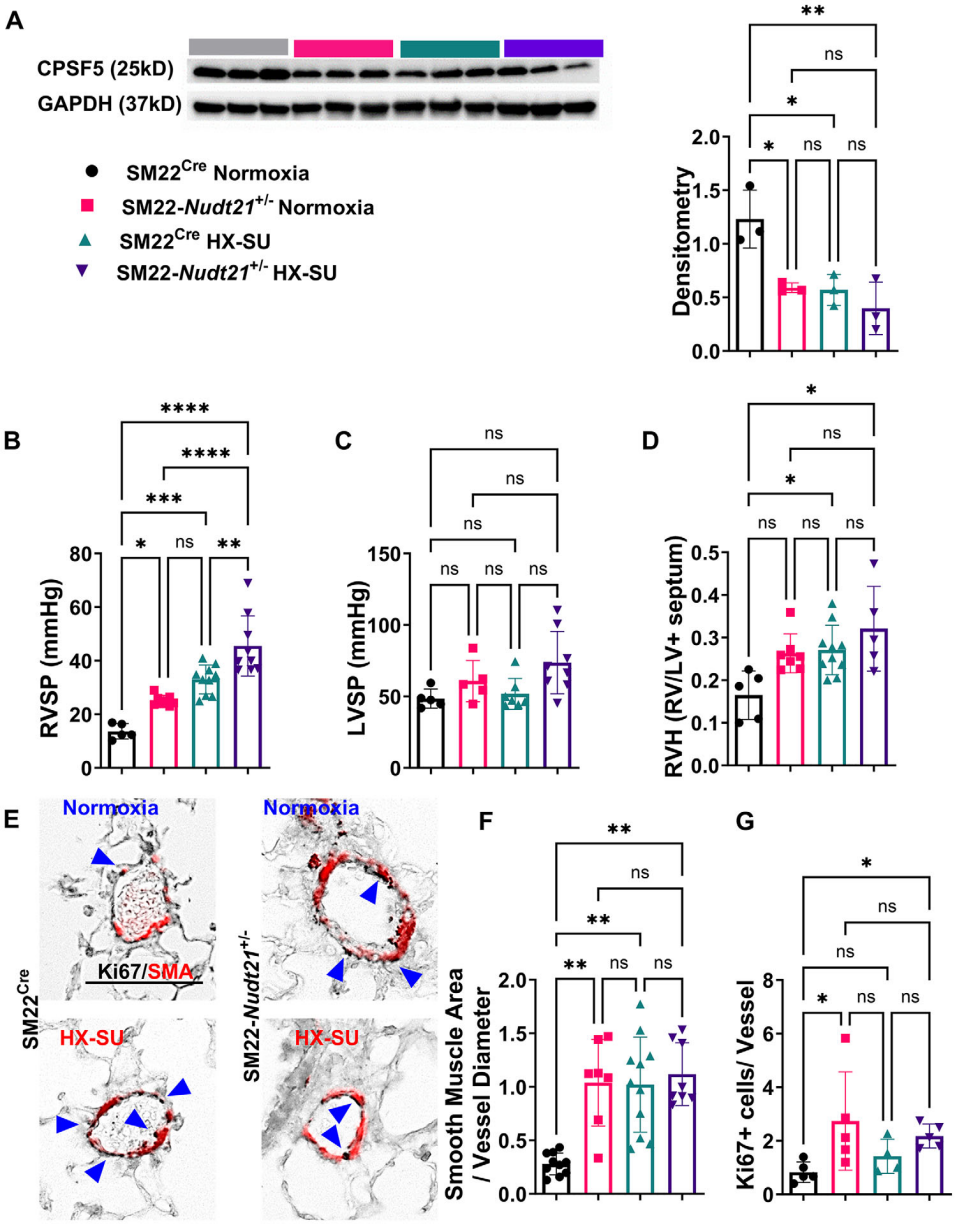
Smooth muscle cell (SMC) *Nudt21* depletion leads to baseline pulmonary hypertension that is augmented in hypoxia–sugen (HX–SU)‐exposed mice. (A) Western blot from isolated PASMC and resulting densitometry quantification for CPSF5 from normoxia or HX–SU‐exposed SM22^Cre^ or SM22*‐Nudt21* ^+/−^ mice. (B) Right ventricle systolic pressure (RVSP) and (C) left ventricle systolic pressure (LVSP) or (D) right ventricle hypertrophy (RVH) from SM22^Cre^ + or SM22*‐Nudt21* ^+/−^ mice exposed to either normoxia or hypoxia–sugen (HX–SU). (E) Dual immunohistochemistry (IHC) for SMA (red) and Ki67 (black) from representative vessels from SM22^Cre^ or SM22*‐Nudt21* ^+/−^ mice exposed to either normoxia or HX–SU, scale bar represents 100 µm, arrows point at Ki67‐positive nuclei. Morphometric quantification identifying a ratio for smooth muscle area to vessel diameter (F) or Ki67‐positive cells per vessel (G) from SM22^Cre^ or SM22*‐Nudt21* ^+/−^ mice exposed to either normoxia or HX–SU. Significance levels ****p < 0.0001, ***0.0001 ≤ *p* < 0.001, ** 0.001 ≤ *p* < 0.01, and *p < 0.05 represent one‐way ANOVA comparisons with the Tukey correction for multiple comparisons. Except for panels C, F, G were comparisons refer to the Kruskal–Wallis nonparametric test with the Dunn correction for multiple comparisons. Biological *N* numbers are as follows: panel A: *N* = 3 for SM22^Cre^ normoxia, *N* = 3 for SM22*‐Nudt21* ^+/−^ normoxia, *N* = 3 for SM22^Cre^ HX–SU, *N* = 3 for SM22*‐Nudt21* ^+/−^ HX–SU. Panel B: *N* = 5 for SM22^Cre^ normoxia, *N* = 8 for SM22*‐Nudt21* ^+/−^ normoxia, *N* = 10 for SM22^Cre^ HX–SU, *N* = 9 for SM22*‐Nudt21* ^+/−^ HX–SU. Panel C: *N* = 5 for SM22^Cre^ normoxia, *N* = 5 for SM22*‐Nudt21* ^+/−^ normoxia, *N* = 7 for SM22^Cre^ HX–SU, *N* = 8 for SM22*‐Nudt21* ^+/−^ HX–SU. Panel D: *N* = 5 for SM22^Cre^ normoxia, *N* = 8 for SM22*‐Nudt21* ^+/−^ normoxia, *N* = 10 for SM22^Cre^ HX–SU, *N* = 5 for SM22*‐Nudt21* ^+/−^ HX–SU. Panel F: *N* = 10 for SM22^Cre^ normoxia, *N* = 7 for SM22*‐Nudt21* ^+/−^ normoxia, *N* = 11 for SM22^Cre^ HX–SU, *N* = 8 for SM22*‐Nudt21* ^+/−^ HX–SU. Panel G: *N* = 5 for SM22^Cre^ normoxia, *N* = 5 for SM22*‐Nudt21* ^+/−^ normoxia, *N* = 4 for SM22^Cre^ HX–SU, *N* = 5 for SM22*‐Nudt21* ^+/−^ HX–SU.

We first assessed whether *Nudt21* depletion in PASMCs influences pulmonary hemodynamics and cell proliferation under normoxia and HX–SU conditions. Mice heterozygous for *Nudt21* in PASMCs were generated using the SM22‐Cre promoter SM22‐*Nudt21^+/−^*). To evaluate the impact of reduced *Nudt21* expression on PH development, SM22‐*Nudt21^±^* and control SM22^Cre^ mice were subjected to chronic HX (10% O_2_ for 4 weeks) with weekly SU5416 injections (HX–SU) or maintained under normoxia (21% O_2_). Western blotting of isolated PASMCs confirmed reduced levels of CPSF5, the protein encoded by *Nudt21*, in SM22‐*Nudt21^±^* mice under both normoxia and HX–SU compared with SM22^Cre^ controls, whereas CPSF5 levels did not decline further with HX–SU exposure (Figure 1A). Complete deletion of *Nudt21* was not viable in SM22Cre mice.

In line with previous studies by our group [46], hemodynamic analysis showed that RVSP was significantly elevated in normoxia‐exposed SM22*‐Nudt21* ^±^ mice compared with normoxic SM22^Cre^ controls (Figure 1B). As expected, HX–SU exposure increased RVSP in SM22^Cre^ mice compared with normoxic controls and this increase was further amplified in HX–SU‐exposed SM22*‐Nudt21* ^±^ mice (Figure 1B). Direct statistical comparison between normoxic and HX–SU‐exposed SM22*‐Nudt21* ^+/−^ mice confirmed a highly significant increase in RVSP and vascular remodeling indices, indicating that *Nudt21* reduction not only elevates RVSP under baseline conditions but also exacerbates the hemodynamic response to HX–SU exposure. No significant changes in LVSP were detected amongst groups (Figure 1C). Right ventricular hypertrophy (RVH), assessed by Fulton index, was increased in HX–SU‐exposed mice (Figure 1D).

Histological analyses revealed evidence of expansion of the tunica media under both conditions. Dual staining for Ki67 and SMA demonstrated increased PASMC proliferation in HX–SU‐exposed SM22^Cre^ and SM22*‐Nudt21* ^±^ mice, with morphometric quantification showing increased SMA deposition in both strains following HX–SU, as well as in normoxic SM22*‐Nudt21* ^+/−^ versus SM22^Cre^ mice (Figure 1E,F). Morphometric quantification of Ki67+/SMA+ signals showed significantly higher proliferative indices in SM22*‐Nudt21* ^+/−^ mice under both normoxia and HX–SU (Figure 1G). Together, these data show that reduced expression of *Nudt21* is sufficient to increase RVSP and PASMC expansion under normoxia and significantly enhances RVSP following HX–SU exposure.

### 2.2 Association of NUDT21 Loss With Altered RUNX1 Expression

**FIGURE 2:**
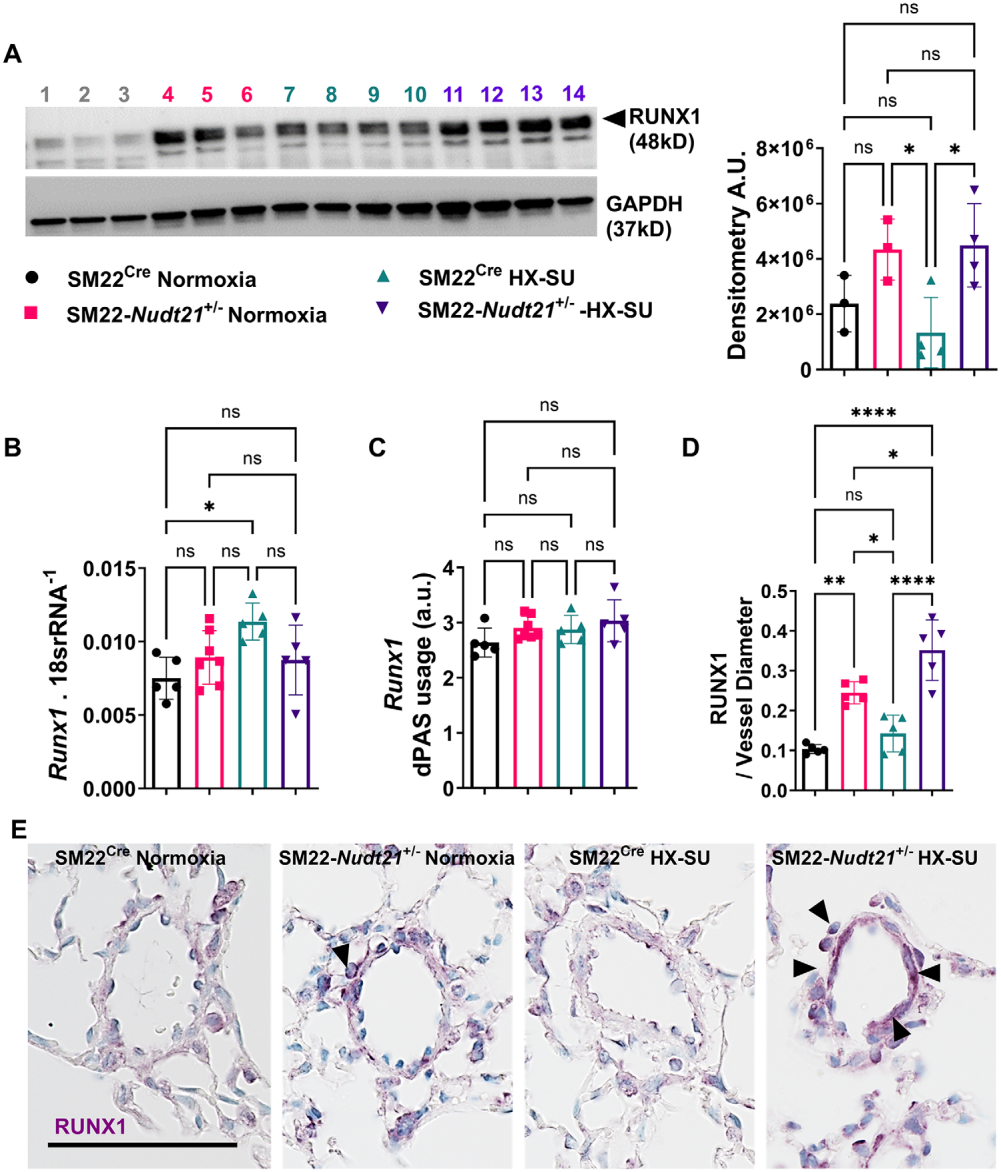
RUNX1 is increased in mouse lungs following smooth muscle depletion of *Nudt21*. (A) Western blot for RUNX1 (top band, denoted by arrowhead) and GAPDH (bottom band) from SM22^Cre^ normoxia (lanes 1, 2, 3), SM22*‐Nudt21* ^+/−^ normoxia (lanes 4, 5, 6), SM22^Cre^ HX–SU (lanes 7, 8, 9, 10), and SM22*‐Nudt21* ^+/−^ HX–SU (lanes 11, 12, 13, 14) and densitometry quantification. *Runx1* gene expression (B) and dPAS usage (C), (D) morphometric quantification identifying a ratio for RUNX1‐positive area to vessel diameter, and (E) representative immunohistochemistry for RUNX1 (purple signals) from from SM22^Cre^ normoxia, SM22*‐Nudt21* ^+/−^ normoxia, SM22^Cre^ normoxia, and SM22*‐Nudt21* ^+/−^ + HX–SU treatment groups. Significance levels ****p < 0.0001, *** 0.0001 ≤ *p* < 0.001, ** 0.001 ≤ *p* < 0.01, and *p < 0.05 represent One‐way ANOVA comparisons with the Sidak correction for multiple comparisons. Arrowheads point at positive RUNX1 areas in vascular structures. The scale bar represents 100 µm. Biological *N* numbers are as follows: Panel A: *N* = 3 for SM22^Cre^ normoxia, *N* = 3 for SM22*‐Nudt21* ^+/−^ normoxia, *N* = 4 for SM22^Cre^ HX–SU, *N* = 4 for SM22*‐Nudt21* ^+/−^ HX–SU. Panels B and C: *N* = 5 for SM22^Cre^ normoxia, *N* = 7 for SM22*‐Nudt21* ^+/−^ normoxia, *N* = 5 for SM22^Cre^ HX–SU, *N* = 5 for SM22*‐Nudt21* ^+/−^ HX–SU. Panel D: *N* = 5 per group.

**FIGURE 3:**
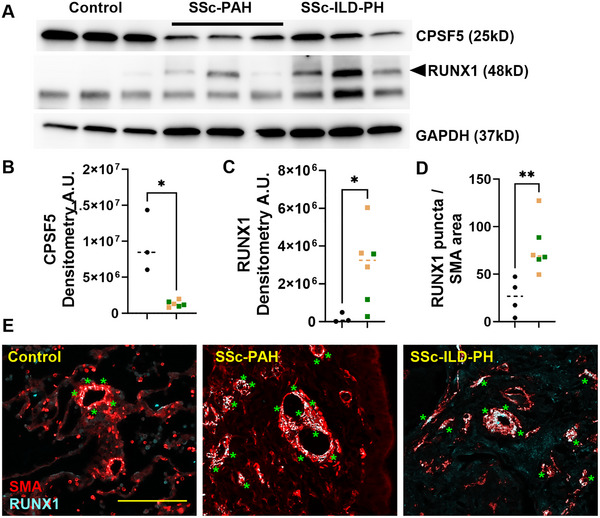
Reduced CPSF5 and elevated RUNXI in SSc‐PH. (A) Western blot from isolated pulmonary arteries from control, SSc‐PAH, and SSc‐ILD‐PH explants for CPSF5 (top band), RUNX1 (middle blot, upper band denoted by the arrowhead), and GAPDH (bottom blot). Densitometry for CPSF5 (B) and RUNX1 (C) from control (black dots) or SSc‐PH explanted lungs (green squatted denote SSc‐PAH and light brown squares denote SSc‐ILD‐PH groups). Morphometric quantification identifying (D) a ratio for RUNX1 puncta to SMA area and (E) representative immunohistochemistry denoting vessels from control (left panel), SSc‐PAH (middle panel), and SSc‐ILD‐PH (right panel) for smooth muscle actin (SMA, red) and RUNX1 (white puncta). The scale bar represents 100 µm and the green asterisk denote SMA cells positive for RUNX1. Significance levels **p 0.001 to 0.01 and *p < 0.05 represent Mann–Whitney comparisons between control and SSc‐PH. Biological *N* numbers are as follows: panels B and C: control (*N* = 3), SSc‐PH (*N* = 6); panel D: control (*N* = 4), SSc‐PH (*N* = 6).

A central process in the pathophysiology of vascular remodeling is PASMC proliferation [1]. Herein, our in vivo data point to reduced levels of *Nudt21* in PASMC, leading to increased cell proliferation and PASMC proliferation under normoxic conditions. To understand the mechanisms that lead to increased vascular cell expansion in SM22‐*Nudt21* ^±^ mice, we performed both DaPARS and QAPA algorithms from RNA‐seq data [47, 48] from HX–SU‐exposed SM22^Cre^ and SM22‐*Nudt21* ^±^ mice. DaPars and QAPA are computational tools that use distinct methods to determine 3′UTR status from standard RNA‐seq datasets (Table S1). Both algorithms revealed increased *Runx1* 3′UTR shortening following *Nudt21* depletion in HX–SU‐exposed SM22‐*Nudt21* ^±^ mice. Since RUNX1 is implicated in vascular wall proliferation and PH [21, 45], we examined whether its expression was altered by *Nudt21* depletion. In line with this, we detected increased RUNX1 protein levels in mouse lung tissues from normoxia or HX–SU‐exposed SM22‐*Nudt21* ^±^ mice (Figure 2A). Gene expression data revealed increased *Runx1* signals only in HX–SU SM22^Cre^ mice (Figure 2B), but unexpectedly, we found no evidence for *Runx1* mRNA 3′UTR shortening among any treatment groups (Figure 2C). Here, we determined distal polyadenylation signal (dPAS) usage is assessed by designing two sets of primers, one that targets the common sequence and another set that targets the distal 3′UTR sequence, allowing us to measure expression levels of both the common and the truncated sequence. Increased expression of RUNX1 was also apparent in remodeled vessels from both normoxia and HX–SU‐exposed SM22‐*Nudt21* ^±^ mice, albeit significantly increased RUNX1 was present in the HX–SU group (Figure 2D,E). Our studies in mice demonstrate that reduced CPSF5 levels in SMCs result in increased RVSP, consistent with elevated SMA deposition and evidence of cell proliferation associated with increased RUNX1 levels. Thus, we next aimed to determine whether CPSF5 and RUNX1 levels were altered in the pulmonary vasculature in SSc‐PH. Using isolated PAs from patients with SSc‐PH, our experiments show reduced CPSF5 levels concomitant with elevated RUNX1 signals in SSc‐PH versus control samples (Figure 3A–C). This is also consistent with dual IHC for RUNX1 and SMA showing increased expression of RUNX1 in remodeled vessels in SSc‐PH versus controls (Figure 3D,E). In line with these studies, RUNX1 levels were highly upregulated in PAs from PAH patients compared with controls (Figure S1A,B). However, paradoxically, gene expression levels for *RUNX1* appeared to be reduced in PAH (Figure S1C), and no evidence of 3′UTR shortening for *RUNX1* was apparent in human PAs (Figure S1D). These results point to RUNX1 in SMCs as a potential mechanism for cell proliferation in PH.

**FIGURE 4:**
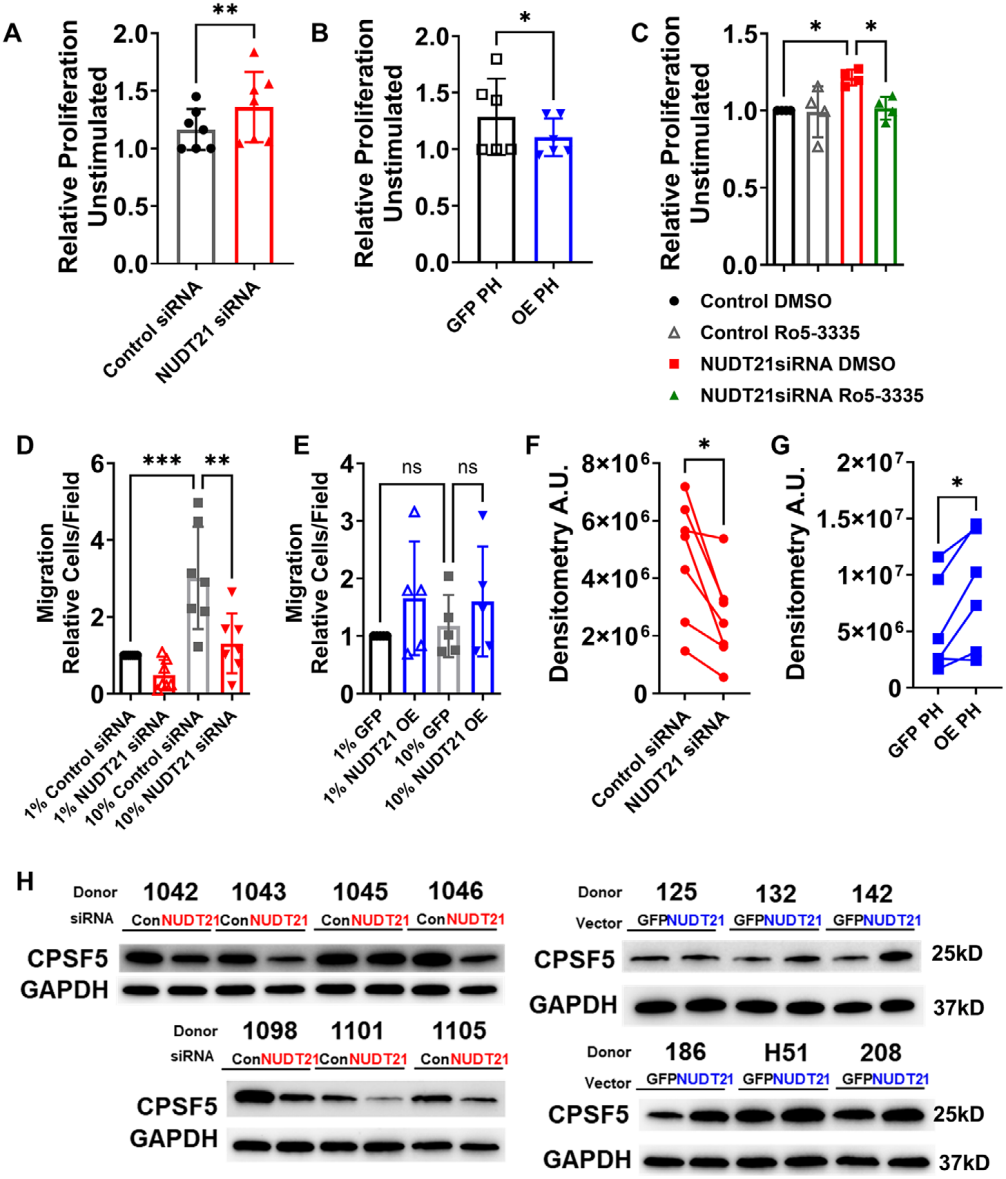
Pulmonary artery smooth cell proliferation is induced by *NUDT21* depletion and is inhibited by pharmacological inhibition of RUNX1. Cell proliferation was determined through the WST1 assay using (A) control pulmonary artery smooth muscle cells (PASMC) treated with either control siRNA or *NUDT21* siRNA or (B) PASMC derived from PAH patients treated with empty vector (EV) or a vector to overexpress *NUDT21*. (C) Cell proliferation determined by the WST1 assay using control PASMCs treated with control siRNA, *NUDT21* siRNA, and DMSO (vehicle for Ro5‐3335) or *NUDT21* siRNA with the RUNX1 inhibitor R05‐3335. Migration assay for control PASMC and treated with 1 or 10% FBS and treated with either control or *NUDT21* siRNA (D) or PASMC derived from PAH patients (E) treated with 1% and 10% FBS an empty vector or a vector overexpressing *NUDT21*. Densitometry for CPSF5 from experiments where *NUDT21* was silenced (F) or where *NUDT21* was overexpressed (G) and corresponding western blots (H) for CPSF5 and GAPDH from control and PAH samples demonstrating successful deletion of NUDT21 in control cells or overexpression of CPSF5 following *NUDT21* overexpression. Significance levels ** 0.001 ≤ *p* < 0.01 and *p < 0.05 represent Wilcoxon matched‐pairs test comparisons for panels A, B, F, and G and one‐way ANOVAs with the Sidak correction for multiple comparisons for panels C, D, and E. Biological *N* numbers are as follows: panels A, D, and F: *N* = 7 per group; panels B, E, and G: *N* = 6 per group; and panel C: *N* = 4 per group.

RUNX1 is known for promoting increased cell proliferation [22, 49]. Thus, we assessed whether *NUDT21* deletion would impact cell turnover. These experiments revealed that *NUDT21* KD in human PASMC increased cell proliferation (Figure 4A). Next, we aimed to determine whether overexpression of *NUDT21* in PAH‐derived PASMC would result in altered proliferation rates. Remarkably, overexpression of *NUDT21* attenuated PAH‐derived PASMC proliferation rates (Figure 4B). To determine whether RUNX1 modulated the increase in cell proliferation induced by *NUDT21* deletion, we used the RUNX1 inhibitor Ro5‐3335 [21]. These experiments revealed that the increase in cell proliferation induced by *NUDT21* KD was reversed by the RUNX1 inhibitor Ro5‐3335 (Figure 4C). We also assessed whether *NUDT21* was able to affect cell migration. In these experiments cell migration induced by 10% fetal bovine serum (FBS) was inhibited following *NUDT21* deletion in control PASMC (Figure 4D), yet *NUDT21* overexpression in PAH‐derived PASMCs did not affect cell migration (Figure 4E). To determine successful KD and overexpression of *NUDT21*, western blots for CPSF5 reveal reduced CPSF5 levels in PAH‐PASMC and increased levels in control PASMC (Figure 4F–H). Here, it is important to note that NUDT21 KD was not effective on control cell 1045 and that CPSF5 overexpression was not significantly elevated in donor 125. Taken together, these results suggest RUNX1 as a mediator that regulates cell proliferation following *NUDT21* depletion; however, the mechanism for this depletion did not seem to involve 3′UTR shortening of RUNX1.

### 2.3 CBFB Upregulation Links With RUNX1 Stabilization

**FIGURE 5:**
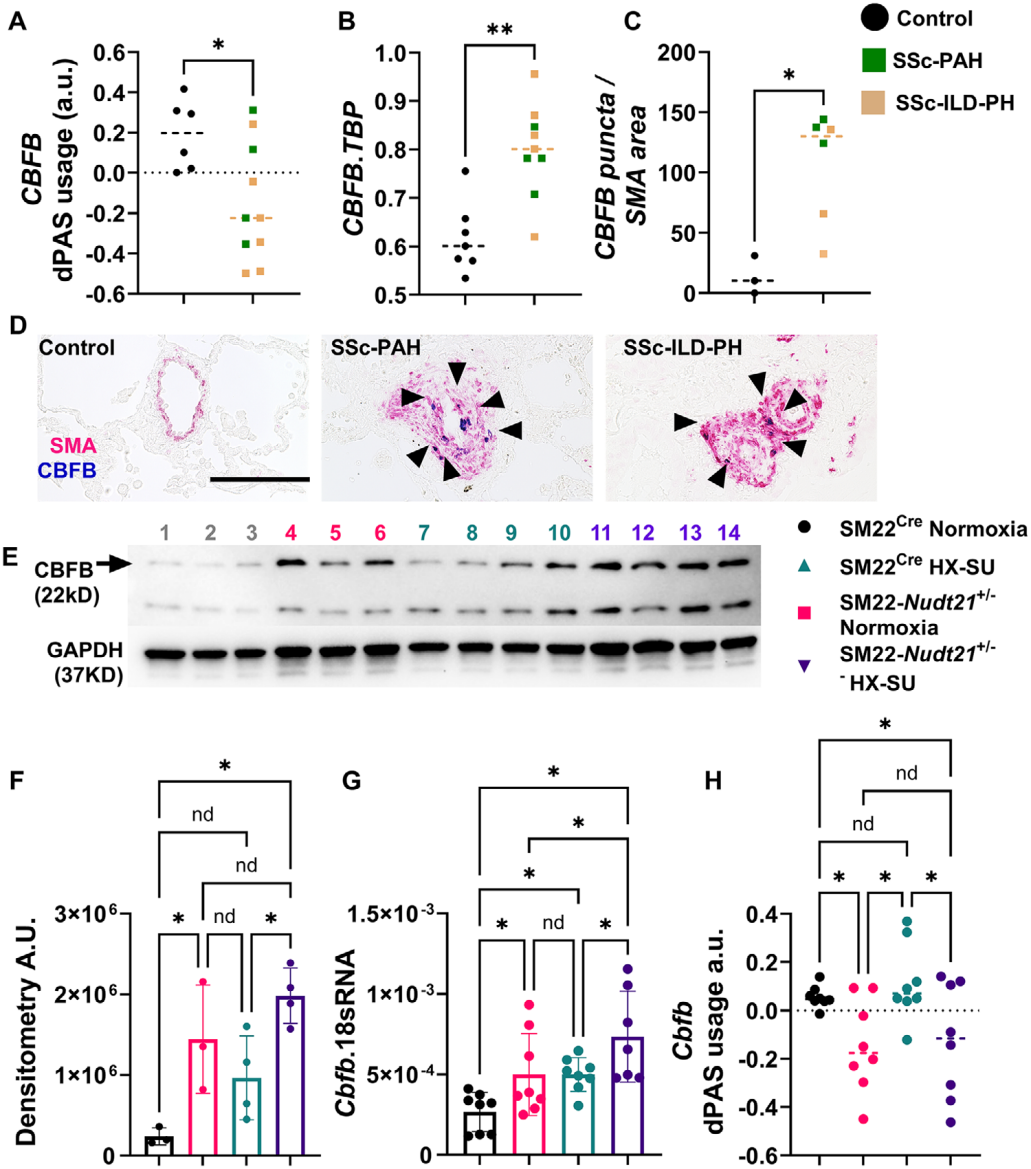
*CBFB* undergoes 3′UTR shortening and increased expression in both SSc‐PH and hypoxia–sugen‐exposed mice. (A) dPAS usage ratio for *CBFB* and (B) *CBFB* gene expression using TATA‐binding protein (*TBP*) as a reference from isolated pulmonary arteries and (C) morphometric quantification of CBFB‐positive puncta relative to SMA area from control from control (black dots) or SSc‐PH explanted lungs (green squatted denote SSc‐PAH and light brown squares denote SSc‐ILD‐PH groups). Significance levels ** 0.001 ≤ *p* < 0.01 and *p < 0.05 represent Mann–Whitney comparisons between control and SSc‐PH for panels A and B and Welch test for panel C. (D) Representative dual immunohistochemistry for vessels from control (left panel), SSc‐PAH (central panel), and SSc‐ILD‐PH (right panel) groups for SMA (pink/magenta signals) and CBFB (blue signals). The scale bar represents 100 µm and arrows point at CBFB cells within the remodeled vessels. (E) Western blot and corresponding densitometry (F) for CBFB (top band, denoted by arrowhead) and GAPDH (bottom band) from SM22^Cre^ normoxia (lanes 1, 2, 3), SM22*‐Nudt21* ^+/−^ normoxia (lanes 4, 5, 6), SM22^Cre^ HX–SU (lanes 7, 8, 9, 10), and SM22*‐Nudt21* ^+/−^ HX–SU (lanes 11, 12, 13, 14). (G) *Cbfb* gene expression and *Cbfb* dPAS usage (H) from SM22^Cre^ normoxia, SM22*‐Nudt21* ^+/−^ normoxia, SM22^Cre^ normoxia, and SM22*‐Nudt21* ^+/−^ + HX–SU treatment groups. Significance levels *p < 0.05 represent one‐way ANOVA comparisons with the Benjamini, Kreiger, and Yekuteli posthoc test for multiple comparisons in panels F, G, and H. Biological *N* numbers are as follows: panel A: control (*N* = 6), SSc‐PH (*N* = 10); panel B: control (*N* = 7), SSc‐PH (*N* = 9); panel C: control (*N* = 3), SSc‐PH (*N* = 6); panel F: SM22^Cre^ normoxia (*N* = 5), SM22*‐Nudt21* ^+/−^ normoxia (*N* = 5), SM22^Cre^ normoxia (*N* = 7) and SM22*‐Nudt21* ^+/−^ + HX–SU (*N* = 4); panels G and H: SM22^Cre^ normoxia (*N* = 8), SM22*‐Nudt21* ^+/−^ normoxia (*N* = 8), SM22^Cre^ normoxia (*N* = 8) and SM22*‐Nudt21* ^+/−^ + HX–SU (*N* = 8).

RUNX1 is regulated by several posttranscriptional mechanisms [50]. Because RUNX1 protein increased while our APA analyses did not reveal robust 3′UTR shortening of *Runx1*, we next investigated posttranscriptional regulators of RUNX1 stability. Here we focused on CBFB based on its well‐established role in stabilizing RUNX1 [51, 52, 53]. Our analytical workflow consisted of a global APA screen followed by short‐listing of candidate transcripts (Table S1), validation of APA status and expression for these candidates, and mechanistic grouping of functionally related targets, including the CBFB–RUNX1 axis. Herein, we detected 3′UTR shortening and increased expression of *CBFB* were present in patients with SSc‐PH (Figure 5A,B). Increased signals for CBFB were also detected in remodeled vessels from SSc‐PAH and SSc‐ILD‐PH individuals (Figure 5C,D). We next detected increased CBFB protein levels in normoxia or HX–SU‐exposed SM22*‐Nudt21* ^±^ mice (Figure 5E,F), these were consistent with increased gene expression levels for *Cbfb* in normoxia or HX–SU‐exposed SM22*‐Nudt21* ^±^ mice (Figure 5G). It is important to note that HX–SU SM22*‐Nudt21* ^±^ mice presented with increased gene but not protein levels compared with normoxia‐treated SM22*‐Nudt21* ^±^ groups (Figure 5F,G). Mouse lung dPAS usage for *Cbfb* showed evidence of 3′UTR shortening in both normoxic or hypoxic SM22*‐Nudt21* ^±^ mice (Figure 5H), no difference was seen among SM22*‐Nudt21* ^±^ groups. In line with this our studies revealed increased protein expression levels for CBFB in remodeled vessels from patients with PAH (Figure S2A,B). This was consistent with evidence of 3′UTR shortening for *CBFB* (Figure S2C) and increased *CBFB* gene expression in isolated PAs (Figure S2D), albeit no changes in protein levels (Figure S2E). Together, our APA analyses revealed reduced dPAS usage in *CBFB*, consistent with 3′UTR shortening. Importantly, this shift in PAS usage correlated with increased *CBFB* transcript levels by quantitative real‐time PCR (RT‐qPCR) and elevated CBFB protein by western blot. These data indicate that CBFB upregulation is associated with 3′UTR shortening following *NUDT21* depletion. In summary, these results point to elevated expression of CBFB, which promotes transcript survival [52] as a mediator that leads to increased RUNX1 levels [51, 52, 53] in experimental PH and in remodeled vessels from patients with distinct presentations of PH.

### 2.4 PTGER3 a Potential Downstream Target for NUDT21 Depletion

To uncover the potential downstream mediators of *Nudt21* reduction under HX, we next examined PTGER3 expression. Our studies reveal that *Nudt21* depletion results in increased and RVSP levels that are much more elevated in HX–SU‐treated SM22*‐Nudt21* ^+/−^ mice compared with HX–SU‐treated SM22^Cre^ mice. Given the importance of HX in the pathogenesis of PH [24, 26], we next aimed to identify how a reduction in *Nudt21* levels in PASMCs altered the response of these cells to HX. Herein, we performed a heat map analysis from RNA‐seq data from isolated PASMCs from HX–SU‐exposed SM22^Cre^ and SM22*‐Nudt21* ^+/−^ mice based on our data demonstrating differences in RVSP between these two groups to identify the potential mechanism for the augmented RVSP response in HX–SU‐exposed SM22*‐Nudt21* ^+/−^ mice (Figure S3). These studies revealed 16 potential gene targets, obtained from edgeR analysis with the cutoff of FDR < 0.05 and fold change > 2 including prostaglandin E receptor 3 (*Ptger3* or *Ep3*).

**FIGURE 6:**
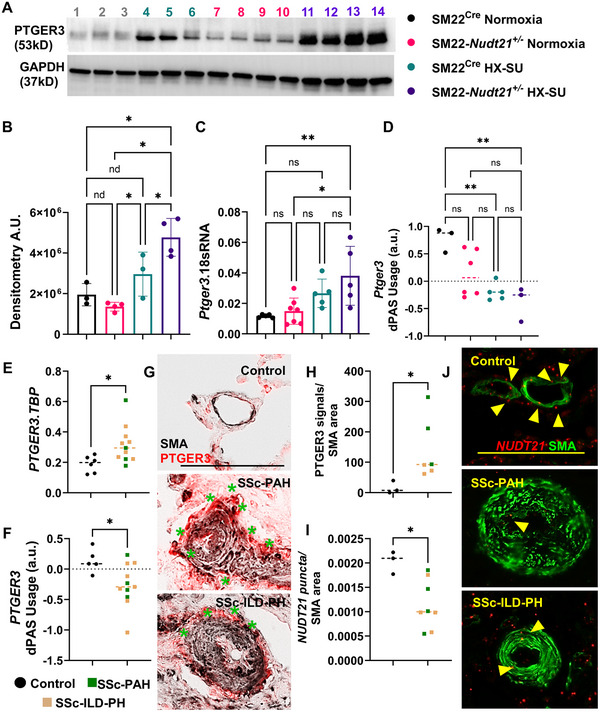
PTGER3 is elevated in remodeled vessels and links with 3′UTR shortening. (A) Western blot for PTGER3 (top band) and GAPDH (bottom band) from SM22^Cre^ normoxia (lanes 1, 2, 3), SM22^Cre^ HX–SU (lanes 4, 5, 6), SM22*‐Nudt21* ^+/−^ normoxia (lanes 7, 8, 9, 10), and SM22*‐Nudt21* ^+/−^ HX–SU (lanes 11, 12, 13, 14), and corresponding densitometry (B). *Ptger3* gene expression (C) and *Ptger3* dPAS usage (D) from SM22^Cre^ normoxia, SM22*‐Nudt21* ^+/−^ normoxia, SM22^Cre^ normoxia and SM22*‐Nudt21* ^+/−^ + HX–SU treatment groups. Significance levels **0.001 ≤ *p* < 0.01 and *p < 0.05 represent one‐way ANOVA comparisons with the Tukey correction for multiple comparisons. Biological *N* numbers are as follows: panel B: SM22^Cre^ normoxia (*N* = 3), SM22*‐Nudt21* ^+/−^ normoxia (*N* = 4), SM22^Cre^ normoxia (*N* = 3) and SM22*‐Nudt21* ^+/−^ + HX–SU (*N* = 4); panel C: SM22^Cre^ normoxia (*N* = 4), SM22*‐Nudt21* ^+/−^ normoxia (*N* = 7), SM22^Cre^ normoxia (*N* = 5) and SM22*‐Nudt21* ^+/−^ + HX–SU (*N* = 5); and panel D: SM22^Cre^ normoxia (*N* = 3), SM22*‐Nudt21* ^+/−^ normoxia (*N* = 6), SM22^Cre^ normoxia (*N* = 5), and SM22*‐Nudt21* ^+/−^ + HX–SU (*N* = 3). (E) *PTGER3* gene expression and (F) dPAS usage ratio for *PTGER3* from isolated pulmonary arteries from control (black dots) or SSc‐PH explanted lungs (green squares denote SSc‐PAH and light brown squares denote SSc‐ILD‐PH groups). (G) Representative dual immunohistochemistry and corresponding morphometric analysis (H) for vessels from control (top panel), SSc‐PAH (central panel), and SSc‐ILD‐PH (bottom panel) for SMA (black/brown signals) and PTGER3 (red signals). The scale bar represents 100 µm and the green asterisk denotes PTGER signals adjacent or within SMA signals. (I) Morphometric quantification of the ratio of *NUDT21* puncta corrected for the SMA area (pixels) from control and SSc‐PH including SSc‐PAH (light brown) and SSc‐ILD‐PH (green). (J) Representative images from stained lung sections for smooth muscle actin (SMA, green) and RNAscope for *NUDT21* (red dots) from control (top), SSc‐PAH (middle) and SSc‐ILD‐PH (bottom) groups. Yellow arrow heads point at *NUDT21* signals in SMA‐stained areas. Scale bar represents 100 µm. Significance levels *p < 0.05 refer to a Mann–Whitney comparison between control and SSc‐PH groups. Biological *N* numbers are as follows: panel E: control (*N* = 6), SSc‐PH (*N* = 11); panel F: control (*N* = 5), SSc‐PH (*N* = 11); and panel H: control (*N* = 3), and SSc‐PH (*N* = 6); panel I: control (*N* = 3) and SSc‐PH (*N* = 8).

In line with these observations, we report increased PTGER3 lung protein levels in HX–SU SM22^Cre^ mice compared with normoxia‐exposed SM22*‐Nudt21* ^+/−^ mice with the highest expression levels in the HX–SU SM22*‐Nudt21* ^+/−^ group (Figure 6A,B). It is important to note that comparisons versus normoxia‐exposed SM22^Cre^ mice only identified increased PTGER3 in the HX–SU SM22*‐Nudt21* ^+/−^ group. We next determined the gene expression of *Ptger3* in our mouse samples using RT‐qPCR assay, revealing increased mouse *Ptger3* only in the HX–SU SM22*‐Nudt21* ^+/−^ group (Figure 6C). Consistently, we found that the *Ptger3* 3′UTR is indeed shortened in HX–SU‐exposed SM22^Cre^ and SM22*‐Nudt21* ^+/−^ groups (Figure 6D). These findings indicate that *Ptger3* upregulation can be driven by hypoxic exposure but is further amplified in the setting of *Nudt21* depletion, consistent with a synergistic effect between *Nudt21* loss and HX.

Next, using isolated PAs from SSc‐PAH and SSc‐ILD‐PH patients, we revealed increased *PTGER3* gene expression (Figure 6E). To identify whether the increased *PTGER3* expression was due to APA, we assessed dPAS usage, which demonstrated *PTGER3* 3′UTR shortening in both SSc‐PAH and SSc‐ILD‐PH samples (Figure 6F). These changes in *PTGER3* transcript levels were consistent with increased PTGER3 protein staining in remodeled vessels, both SSc‐PAH and SSc‐ILD‐PH detected by IHC and assessed morphometrically (Figure 6G,H). These were consistent with reduced *NUDT21* in remodeled vessels from patients diagnosed with SSc‐PAH and those with SSc‐ILD‐PH as shown histologically (Figure 6 I). Morphometric quantification of *NUDT21* signals revealed reduced expression in remodeled vessels in SSc‐PAH and SSc‐ILD‐PAH (Figure 6J). In line with increased expression of PTGER3 in SSc‐PH samples, we detected elevated PTGER3 in remodeled vessels in PAH by IHC and corresponding morphometric quantification (Figure S4A,B). This was in line with increased *PTGER3* gene expression levels and evidence of 3′UTR shortening for *PTGER3* in PAH samples (Figure S4C,D). The increased levels of 3′UTR shortening and mRNA were consistent with the increase in PTGER3 protein levels in isolated PAs from PAH samples and corresponding densitometry (Figure S4E). These observations align with previous studies from our group showing reduced *NUDT21* levels in vessels from PAH patients [35] and point to loss of *NUDT21* as a potential mechanism that promotes vascular remodeling. Herein, we demonstrate that PTGER3 is a downstream target for NUDT21 that contributes to the development of vascular remodeling in both SSc‐PH, including SSc‐PAH and SSc‐ILD‐PH, and in PAH.

### 2.5 miR‐3163 Target *NUDT21* Are Elevated in PH

**FIGURE 7:**
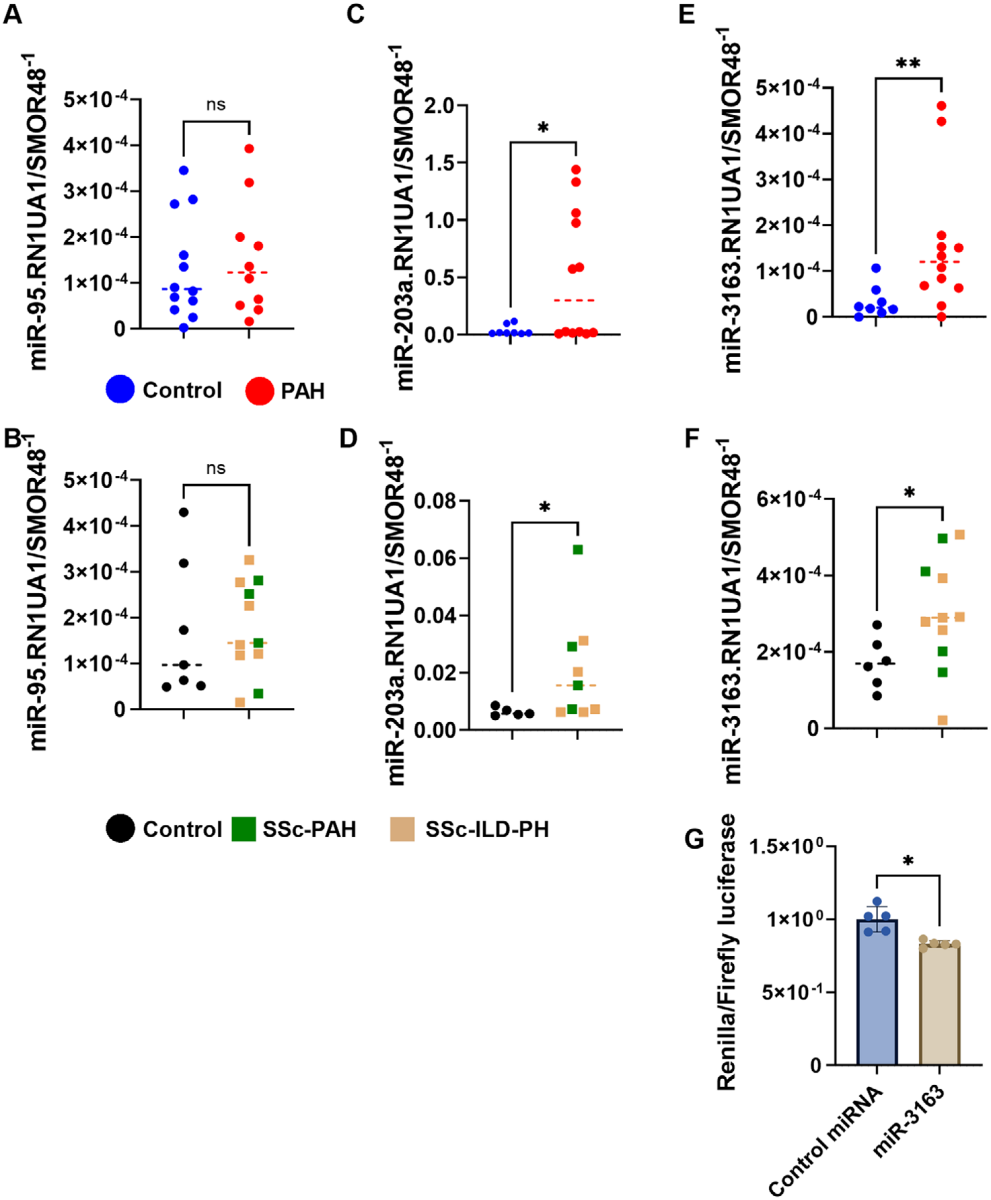
miRs targeting *NUDT21* are upregulated in both PAH and SSc‐PH. Expression levels for miR‐95 from control versus PAH (A) and control versus SSc‐PH (B); miR‐203a from control versus PAH (C) and control versus SSc‐PH; (D) and miR‐3163 from control versus PAH (E), control versus SSc‐PH (F). Data are presented as gene expression normalized to the geometric mean of RN1UA1 and SMOR48). MiR‐mimic experiment for miR‐3163 targeting NUDT21 plasmid. Significance levels ** 0.001 ≤ *p* < 0.01 and *p < 0.05 represent Mann–Whitney comparisons between control and SSc‐PH (green squares denote SSc‐PAH and light brown squares denote SSc‐ILD‐PH groups) or control miR (scramble [scr]) versus miR‐3163 mimic. Biological *N* numbers are as follows: panel A: control (*N* = 12), PAH (*N* = 10); panels C and E: control (*N* = 8), PAH (*N* = 12); panel B: control (*N* = 7) and SSc‐PH (*N* = 11); panel D: control (*N* = 5) and SSc‐PH (*N* = 9); panel F: control (*N* = 6), and SSc‐PH (*N* = 11); panel G: Scr miR (*N* = 5), and miR‐3163 mimic (*N* = 5).

Our studies have demonstrated how *NUDT21* depletion results in increased expression of PTGER3, a mediator involved with increased vascular tone in PAH [19] through direct 3′UTR shortening of this transcript. In addition, we have shown how *NUDT21* depletion leads to 3′UTR shortening *CBFB*, an RNA chaperone that increases the mRNA survival of RUNX1 [51, 52, 53]. However, the mechanisms that lead to *NUDT21* downregulation are not known. Herein, we evaluated the expression of miRs associated with *NUDT21* depletion, determined by published studies [54] and through targetscan analysis. These studies performed in isolated PAs revealed no significant differences in miR‐95 expression in PAH or SSc‐PH, including SSc‐PAH and SSc‐ILD‐PH, compared with their respective controls (Figure 7A,B). However, increased miR‐203a and miR‐3163 were elevated in either PAH or SSc‐PH samples compared with their respective controls (Figure 7C–F). Mir mimic studies revealed that miR‐31363 is able to target *NUDT21* for degradation as demonstrated in our in vitro studies (Figure 7G). Taken together, these results point to miR‐3163 as mediator that can deplete *NUDT21* expression in PH.

## 3 Discussion

PH remains a challenging condition associated with significant morbidity and mortality, particularly in SSc, where multiorgan involvement complicates management [55, 56, 57]. Although survival has improved in SSc‐PAH, outcomes for Group 3 PH (SSc‐ILD‐PH) have stagnated over the past two decades [56]. Current therapies largely target vasodilation [58, 59] and are insufficient to address vascular remodeling, a hallmark of PH [59]. While Treprostinil has recently been approved for Group 3 PH [60], it is not yet recommended in SSc‐PH despite evidence that the prostacyclin pathway contributes to disease [61, 62]. These limitations underscore the need for therapies targeting upstream mechanisms of remodeling.

Here, we identify *NUDT21*/CPSF5 depletion as a unifying upstream event that promotes features of vascular remodeling in both PAH and SSc‐PH. We demonstrate loss of CPSF5 in remodeled vessels from patients, and in mice, partial *Nudt21* deletion in PASMCs increased PASMC proliferation and tunica media thickening under normoxia and further exacerbated RVSP elevation with HX–SU exposure. Importantly, these changes were associated with 3′UTR shortening of *PTGER3* and *CBFB*, linking APA remodeling to cell proliferation and thickening of the tunica media, features of vascular remodeling.

Our results expand prior work showing APA‐mediated regulation of *HAS2* [35 *]* by demonstrating that CPSF5 depletion also influences *PTGER3* and *CBFB*. Elevated PTGER3 protein levels correlated with *Nudt21* loss under HX, suggesting that CPSF5 depletion amplifies HX‐induced signals. PTGER3 had previously been implicated in PH through TGFβ signaling [19], but the upstream cause of its elevation was unknown. Our data now connect this increase directly to APA dysregulation. Notably, CPSF5 levels were not further reduced with HX–SU, suggesting that context‐specific APA patterns rather than additional depletion account for the heightened PTGER3 expression in SM22*‐Nudt21* ^+/−^ mice under HX

Similarly, we found increased CBFB expression, which protects RUNX1 from degradation [51, 52, 53]. While QAPA/daPARS predicted *RUNX1* 3′UTR shortening, validation was inconsistent across isoforms. Instead, our findings support a model where APA of *CBFB* indirectly stabilizes RUNX1, consistent with prior reports identifying RUNX1 as a mediator of vascular remodeling in PH [21, 45]. Thus, while our data are consistent with a model in which CPSF5 depletion leads to 3′UTR shortening of *CBFB*, leading to increased CBFB expression and subsequent stabilization of RUNX1. We acknowledge that the direct role of CBFB in mediating CPSF5 depletion‐induced changes in RUNX1 has not been demonstrated here. Rather, our findings, together with prior studies, suggest this mechanism as a plausible explanation for the observed increase in RUNX1 levels following CPSF5 depletion. Collectively, these results position reduced *NUDT21*‐mediated APA as an upstream event that could contribute to increased RUNX1 levels though *CBFB* regulation.

An additional unexpected observation was that CPSF5 depletion enhanced proliferation but inhibited migration of PASMCs. While proliferation and migration often co‐occur, they can be uncoupled. For example, Paulin et al. emphasized that motility of PASMCs can be selectively altered in PH, independent of proliferative responses [63]. Similarly, reviews of VSMC phenotype switching describe synthetic versus contractile states in which proliferative capacity may be heightened while migratory potential is reduced [64]. By contrast, many reports describe coactivation of both proliferation and migration under pathological stimuli, such as RAS protein activator‐like 2 in PAH [65]. Based on our transcriptomic data, we propose that CPSF5 depletion remodels APA of transcripts to favor cell cycle activation while concurrently dampening expression of cytoskeletal and adhesion regulators, leading to a hyperproliferative but less motile PASMC phenotype. This divergence, although unusual, may bias SMCs toward clonal expansion within the vessel wall and thus contribute to vascular remodeling in PH.

Reduced *NUDT21* expression has been reported in PAH [35], lung fibrosis [66], and dermal fibrosis [67], underscoring its role as a common driver of pathological remodeling. We extend this by identifying miR‐3163 as candidate regulators of *NUDT21*. Prior work showed that TGFβ signaling induces miR‐203a in lung fibrosis [54]; we propose a similar mechanism in PH where elevated miR‐3163 could suppress *NUDT21*, amplifying APA dysregulation in PH.

We acknowledge that APA changes do not always directly predict protein expression due to additional layers of posttranscriptional regulation. Furthermore, while our data support CBFB as a mediator of RUNX1 stabilization, this link was not directly tested, yet it has been identified by several studies [51, 52, 53]. Future studies employing CBFB knockdown or overexpression in PASMCs will be needed to confirm this relationship.

Although our study integrates in vivo, ex vivo, and in vitro approaches to establish a role for *NUDT21*/CPSF5 in pulmonary vascular remodeling, several limitations should be acknowledged. First, while we demonstrate smooth‐muscle‐specific *Nudt21* depletion, off‐target sm22^Cre^ activity in other lineages cannot be excluded. Second, mechanistic links between APA changes in CBFB and RUNX1 stabilization were inferred from expression and prior literature rather than confirmed by direct binding or reporter assays. Third, although our PASMC and human explant data strongly support a role for PTGER3 and RUNX1, additional lineage‐tracing and rescue studies will be required to establish causality. Finally, our miRNA findings identify miR‐3163 as a potential regulator of NUDT21, but in vivo validation using antagomirs or conditional overexpression remains to be performed. Future studies addressing these aspects will further clarify the contribution of APA dysregulation to vascular remodeling in PH.

## 4 Materials and Methods

### 4.1 Ethical Approval

All animal experiments were reviewed and approved by the Animal Welfare Committee (AWC) of the University of Texas Health Science Center at Houston, protocol AWC‐16‐0060, AWC‐19‐0029 AWC‐22‐0028.

The use of human material for this study was reviewed by the University of Texas Health Science Center at Houston Committee for the Protection of Human Subjects (Institutional Review Board no. HSC‐MS‐08‐0354 and HSC‐MS‐15‐1049). Experiments utilizing human material was performed following the Declaration of Helsinki and informed consent was obtained from each participant. Written informed consent was obtained from all participants. Deidentified lung explant tissue from patients with a diagnosis of pulmonary arterial hypertension (PAH) or SSc with either PAH or ILD with PH corresponding deidentified clinical parameters were obtained from the Houston Methodist and Memorial Hermann Hospitals (Houston, TX). PAH, and ILD was diagnosed by board‐certified pulmonologists upon admittance for lung transplantation. Control lung tissue was obtained from lungs that were declined for transplantation but had no chronic pulmonary disease or contusion. Healthy control lung tissue was obtained from the International Institute for the Advancement of Medicine (Edison, NJ). Lungs were processed for formalin fixed paraffin embedded (FFPE) and flash frozen preparations as previously described [26].

### 4.2 Animals

Male and female 8–10 week old mice weighing 20–22 g, were used in this study. Mice generated for this study included the development of a SMC‐specific NUDT21 depleted mice using the Cre–Lox sytem under the control of the transgelin (Tagln also known as smooth muscle 22 [SM22] alpha) promoter as previously described [68]. These mice were bred with *Nudt21*‐loxP/loxP mice [66] to generate SM22‐*Nudt21* ^±^ mice. Intriguingly, no homozygous SM22‐*Nudt21* mice could be generated. Mice were housed in ventilated cages with micro isolator lids with food and water ad libitum. Mice were kept on a 12‐h light–dark cycle, at a temperature of 22°C. Animal care was conducted in accordance with institutional and U.S. National Institute of Health guidelines. All studies were reviewed and approved by the University of Texas Health Science Center at Houston Animal Welfare Committee.

*Experimental Model of PH*. *Chronic HX–SU PH Model*: Mice were exposed to chronic isobaric HX for 28 days. Briefly, animals in open cages were placed in isobaric plastic chambers (Biospherix A‐Chamber, Lacona, NY) and exposed to inspired O_2_ fraction of 10%. The oxygen levels were maintained using an atmospheric regulator from OKO Labs (Pozzuoli, NA, Italy). The vascular endothelial growth factor receptor antagonist, Sugen5416 (20 mg/kg) was injected IP once‐weekly in mice during exposure to 10% oxygen for 28 days [35, 37]. After assessment of PH as described below, rodents were euthanized by anesthetic overdose followed by cervical dislocation and bilateral thoracotomy. Lungs were perfused through the RV and the left lobe excised for histology.

*Assessment of PH*. Right ventricular systolic pressure (RVSP) was determined by transdiaphragmatic cardiac puncture as previously described [37]. Briefly mice were anesthetized with a single dose of 0.5 mg g^−1^ of 10% tribromoethanol solution (TBE; a mixture of tert‐amyl alcohol and 2–2‐2 tribromoethanol; Sigma–Aldrich) to induce a surgical plane of anesthesia. 2,2,2 Tibromoethanol and tert‐amyl alcohol are purchased at 99% purity to make TBE. No pharmaceutical grade TBE is available. Proper anesthetic depth was confirmed by visualizing pink ears and mucous membranes, the absence of blinking when the eyelid was touched, and the absence of foot withdrawal when the toes or feet were pinched. Once the proper anesthetic depth was confirmed with the aforementioned methods, a tracheotomy was performed by cutting an opening of 2–3 mm in the trachea and an 19‐gauge tubing adaptor was used to cannulate the trachea and attached to a mouse ventilator. We selected TBE as it has been shown to produce less bradycardia and left ventricle loading compared with ketamine/xylazine combinations [69] although TBE and isoflurane have a similar cardiovascular profile [70] isoflurane has been shown to depress the cardiovascular system at higher doses and produce an initial increase in heart rate lasting up to 20 min in mice [71]. For these reasons we selected TBE. Data were captured in LabChart (AD Instruments, Colorado Springs, CO). A 30‐s interval comprising eligible parameters for HR (300–450 bpm), end‐diastolic pressure (≤5 mmHg), and systolic variation (<10%) were selected for analysis. RVSP measurements were excluded if readings fell outside of these limits. Mice were euthanized following an overdose of TBE (5 mg g^−1^ of 10% TBE) followed by cervical dislocation.

*Human PA Isolation*. PAs were identified visually and dissected free from explanted lung tissue, beginning with a midline transverse cut, separating proximal and distal sections for each lobe. Isolated arterial segments ranged in diameter from 0.5 to 4 mm. The PAs were flash frozen in liquid nitrogen and pulverized using the 6875 CryoMill (SPEX SamplePrep). Demographic and hemodynamic data are summarized in Table S2.

*Isolation of PASMC*. Human and mouse PASMC were isolated using an adapted protocol from Lee et al. [72]. Distal PAs are dissected from the explanted lung, with the adventitia removed manually and the vessels cut longitudinally. The vessels are digested using elastase (LS002290; Worthington) and collagenase type II (LS004174; Worthington) for 1.5 h at 37°C. Debris are filtered and the cells centrifuged and cultured in SMCM (1101; ScienceCell). Cells are analyzed initially by confirming stellate morphology. Confirmation though staining shows positive for alpha‐smooth muscle actin (SMA) (A2647; Sigma), desmin (ab15200; abcam), transgelin (EPR11995 abcam) and negative for von Willebrand Factor VIII (NB600‐586; Novus Biologicals).

*Cell Culture*. Cells were cultured in smooth muscle growth media (SmGM‐2; Lonza) supplemented with penicillin/streptomycin, 2% FBS, and smooth muscle growth supplement (Lonza). Control and PAH‐derived HPAMSCs lines were obtained from the UTHealth Pulmonary Center of Excellence Biobank. Cells between passages 4 and 8 were used in all experiments

*Measurement of Proliferation with WST‐1*. PASMCs were grown to 50% confluence in 96‐well plates and then placed in starvation media for 48 h following the transfection with siRNA or expression plasmids. The cells were then stimulated with PDGF‐BB (50 nM PDGF‐BB; R&D Systems, Minneapolis, MN) for 48 h. Ten microliters of WST‐1 reagent (Roche, Basel, Switzerland) was added for 4 h at 37°C and 5% CO_2_. The plates were read at absorbance of 440 nm and a reference wavelength of 600 nm.

*Transfection of Cells*: Plasmids were transfected with jetPRIME (Polyplus, Illkirch, France) by preincubating plasmid and jetPRIME reagent for 10 min, 0.1 µg plasmid for 96‐well plate or 0.5 µg for 24‐well plates. The mixture was added to the cells overnight. siRNA transfection utilized INTERFERin (Polyplus) preincubation with 1 nM siRNA and reagent for 10 min and then incubated with the cells overnight.

*RT‐qPCR*. Flash frozen lungs were pulverized with a mortar and pestle. RNA was extracted with Triazole and chloroform. RNA was obtained by addition of Cotrimoxazole lysis reagent (QIAzole; QIAGEN, Hilden, Germany) followed by extraction with chloroform added in a 1:5 ratio. Following precipitation with 100% ethanol, samples were passed through an RNA‐binding spin column (miRNeasy; QIAGEN). The column was washed and mRNA eluted with water. Total RNA was quantified by A260/280 ratio via NanoDrop. cDNA synthesis was performed by reverse transcription reaction (iScript; BioRad) using 2000 ng of template. For RT‐qPCR reactions, between 20 and 40 ng of cDNA were utilized per well and amplified with Sybr‐Green primer pairs listed above. No‐template controls were included in each experiment. Relative expression against the 1*8S* ribosomal RNA or TBP was determined via the double delta C_t_ method. Fluorometric amplification signal was monitored in real time with a CFX Opus 384 Real‐time PCR system (BioRad, Hercules, CA). Primers for miR‐95, miR‐203a, miR‐3163 as well as RN1UA1 and SMOR48 were obtained from Horizon Discovery (Cambridge UK). Results from miR expression were normalized to the geometric mean of RN1UA1 and SMOR48 expression. The list of primers used is included in Table S3.

*Analysis of 3′UTR Length*. Candidate genes for validation were selected based on (i) significant APA changes identified by QAPA/daPARS, (ii) prior evidence linking the gene to PASMC proliferation or vascular remodeling in PH, and (iii) availability of reliable reagents for validation. *Runx1* fulfilled these criteria and was therefore prioritized for follow‐up analysis. To detect 3′UTR shortening, we evaluated dPAS usage by RT‐qPCR. We used siRNA to silence *NUDT21* (MISSION siRNA SASI_HS01_0014687 at 50 ng/mL; Sigma) versus scrambled siRNA for the control condition. Two pairs of primers were designed for each target gene; the first pair targeting the open reading frame to yield the total transcript level, and the second targeting 3′UTR sequences just 5’ of dPAS to detect long transcripts spanning the dPAS. For each gene, the relative abundance of long to total transcripts was quantified as

$$\Delta Ct=Ct_{\mathrm{total}}\;-Ct_{\mathrm{distal}}$$

where Ct denotes the PCR cycle threshold. Data were presented as fold change normalized to control as previously described [35]. The list of primers used is included in Table S3.

Underlines sequences denotes primers that were also used for total gene expression.

### 4.3 miRNA Regulation Assay

Dual‐Luciferase plasmid(psiCheck2) was used based on prior studies [54] with the human *NUDT21* 3′‐UTR behind the *firefly* sequence and using the *Renilla* luciferase as an internal control. miRNA‐3163 mimic was acquired from Horizon Discovery Biosciences Limited (Cambridge UK), with the sequence 5′‐PUAUAAAAUGAGGGCAGUAAGAC‐3′. The plasmid was cotransfected with the miRNA mimic using Lipomaster 3000 reagent (Vazyme, Nanjing, PRC). After 18 h the luciferase activity was read with the Dual‐Glo Luciferase kit (Promega, Madison, WI) with a Tecan Infinite 200 Pro.

*Immunohistochemistry and Vascular Morphometry*. Formalin‐fixed paraffin‐embedded lungs were sectioned and processed for immunohistochemistry as previously described [39, 73]. The lung sections were stained using antibodies listed in Table S4.

*Microscopy, Image Processing, and Quantitation of Vascular Remodeling*: For detection of alpha SMA, mouse lung slides were imaged at 20× brightfield (Keyence BZ‐X800; Itasca, IL).

### 4.4 Western Blots

Pulverized lung is dissolved in RIPA buffer (Thermo Scientific, Rockford, IL) containing 1 mM protease and phosphatase inhibitor (Sigma–Aldrich). Twenty micrograms of protein was mixed with 6X SDS‐Sample buffer (Boston BioProducts) and loaded into 4–12% Mini‐Protean TGX gels (Bio‐Rad, Hercules, CA). These gels were then transferred to PVDF membrane (GE Healthcare, Piscataway, NJ). Membranes were blocked with 5% milk in TBS‐T (Sigma–Aldrich) for 1 h and incubated with the primary antibody ON at 4°C. Secondary antibodies were incubated for 1 h at RT and the blots were visualized with Clarity ECL (Bio‐Rad). Densitometry was performed with the Bio‐Rad Image Lab software. The list of antibodies used is included in Table S4.

*Data and Statistical Procedures*. Data were analyzed with Prism 10 software (GraphPad, La Jolla, CA). For all rodent experiments, we used four to eight independent animals per group, affording 80% power to exclude an effect size of at least 1.6 assuming a Gaussian distribution. All investigators were blinded to the rodent genotype, which was retroactively assigned by genotyping at the time of statistical analysis. In vitro experiments were carried out with between four and six unique cell lines on at least two separate occasions. All experiments were tested for normality using the D'Agostino & Pearson and Shapiro‐Wilk tests. If any data failed the normality test, a nonparametric statistical test was performed to analyze data. Parametric tests for experiments with more than two groups, we utilized parametric two‐way ANOVA testing with either a Tukey or Sidak posthoc test. The Kruskal–Wallis test with the Dunn's correction was the selected nonparametric test. For unpaired data consisting of two groups, we utilized the parametric two‐tailed Welch's *t*‐test, accounting for unequal variances and/or sample sizes. For paired data consisting of repeated measures on the same group, we utilized parametric two‐tailed student's paired *t‐*test assuming uniform variances. The Mann–Whitney *U*‐test was used as a nonparametric test involving two groups only. For data pertaining to repeated observations of the same group subjects across time, such as before and after treatment, we utilized one‐way repeated measures ANOVA. Statistical significance was defined as *p* < 0.05. Outliers were excluded only if they fulfilled the ROUT test and are reported in the corresponding figure legend whenever applicable.

### 4.5 Preprocessing and Differential Analysis of RNA‐Seq Data

The raw reads obtained from RNA‐Seq were mapped to mouse reference genome mm10 using STAR [74] with default parameters. Gene expression level was calculated as count data with htseq‐count [75]. R edgeR [76] was used to obtain the differentially expressed genes between two different conditions with FDR cutoff of 0.05 and fold change cutoff of 2.

## Author Contributions

Study design, collected data, performed experiments, analyzed data, and wrote the paper: HKQ and SDC. Study design and analysis of data: EJW, LZ, WJZ, and SA. Collected data and performed experiments: TWM, RG, TCJM, CW, JT, and NW. Interpreted results: BA, BP, and HKY. Provided research materials: EES, HJH, and RH. All authors have read and approved the final manuscript.

## Funding

This study was funded by the NHLBI: R01HL138510 R01HL157100 to HKQ and the William S. Kilroy, Sr. Distinguished University Chair in Pulmonary Disease to HKQ. This work is also partly supported by DoD W81XWH‐22‐1‐0164 and NIH 1 UL1 TR003167 01 to WJZ.

## Conflicts of Interest

The authors declare no conflicts of interest.

## Ethics Statement

Animal experiments were reviewed and approved by the AWC of the University of Texas Health Science Center at Houston, protocol AWC‐16‐0060, AWC‐19‐0029 AWC‐22‐0028. The use of human material for this study was reviewed by the University of Texas Health Science Center at Houston Committee for the Protection of Human Subjects (Institutional Review Board no. HSC‐MS‐08‐0354 and HSC‐MS‐15‐1049).

## Supporting information




**Supporting Table 1**: Summary of alternative polyadenylation (APA) analysis results.
**Supporting Table 2**.
**Supporting Table 3**: Mouse and Human primers used in study
**Supporting Table 4**: Antibodies utilized for research.
**Supporting Figure 1**: RUNX1 is elevated in remodeled vessels from patients with PAH.
**Supporting Figure 2**: *CBFB* undergoes 3′UTR shortening and increased expression in PAH.
**Supporting Figure 3**: Heat map identifying altered gene expression following hypoxia–sugen exposure in mice with reduced smooth muscle *Nudt21 expression*.
**Supporting Figure 4**: *PTGER3* undergoes 3′UTR shortening and increased expression in PAH.

## Data Availability

The authors have nothing to report.
