# Supplementary material for: Cleavage and Polyadenylation Specificity Factor Subunit 5 Regulates Pulmonary Artery Smooth Muscle Expansion and Hypoxic Response
Source: MedComm (2020). 2026 Feb 3;7(2):e70610. doi: 10.1002/mco2.70610 (PMC12868934; doi:10.1002/mco2.70610)
Supplement: Supplementary file 1 — Supporting Table 1: Summary of alternative polyadenylation (APA) analysis results. Supporting Table 2. Supporting Table 3: Mouse and Human primers used in study Supporting Table 4: Antibodies utilized for research. Supporting Figure 1: RUNX1 is elevated in remodeled vessels from patients with PAH. Supporting Figure 2: CBFB undergoes 3′UTR shortening and increased expression in PAH. Supporting Figure 3: Heat map identifying altered gene expression following hypoxia–sugen exposure in mice with reduced smooth muscle Nudt21 expression. Supporting Figure 4: PTGER3 undergoes 3′UTR shortening and increased expression in PAH. [file MCO2-7-e70610-s001.docx]

### Cleavage and polyadenylation specificity factor subunit 5 regulates pulmonary artery smooth muscle expansion and hypoxic response

###

### ^1^Scott D. Collum, ^2^Lisha Zhu ^1^Tingting W Mills, ^1^Rene Girard, ^1^Jamie Tran, ^1^Tinne CJ Mertens, ^1^Cory Wilson, ^3^Nancy Wareing, ^4^Erik E. Suarez, ^4^Howard J Huang, ^5^Rahat Hussain, ^5^Bindu Akkanti, ^2^Wenjin J Zheng, ^6^Hari K Yalamanchili,^5^Bela Patel, ^7^Eric J Wagner, ^8^Sandeep Agarwal and ^1, 5^Harry Karmouty-Quintana

^1^Department of Biochemistry and Molecular Biology, McGovern Medical School, University of Texas Health Science Center Houston, TX 77030, USA

^2^Data Science and Informatics Core for Cancer Research, McWilliams School of Biomedical Informatics, University of Texas Health Science Center Houston, TX 77030, USA

^3^Department of Internal Medicine, Emory University School of Medicine, Atlanta, GA, 30322, USA

^4^DeBakey Heart and Vascular Center, Houston Methodist Hospital, Houston, TX 77030, USA.

^5^Division of Pulmonary, Critical Care and Sleep Medicine, Department of Internal Medicine, McGovern Medical School, University of Texas Health Science Center Houston, TX 77030, USA.

**^6^**Department of Department of Pediatrics, Baylor College of Medicine, Houston, TX 77030, USA

**^7^**Department of Biochemistry and Biophysics, The University of Rochester Medical Center, Rochester, NY 14642

**^8^**Section of Immunology, Allergy and Rheumatology, Department of Medicine, Baylor College of Medicine, Houston, TX 77030, USA

**Supplementary Data**

**Table S1**

| *Gene* | H1_median_PAU | H2_median_PAU | diffPAU | UTR_change | H1_Mean_PDUI | H2_Mean_PDUI | PDUI_diff | adjusted_P_val | UTR_change2 |
| --- | --- | --- | --- | --- | --- | --- | --- | --- | --- |
| *Cks1b* | 45.855 | 67.587 | 21.732 | Shortening | 0.813333333 | 0.606666667 | 0.20666667 | 2.46E-21 | Shortening |
| *Tgfbr2* | 15.477 | 45.014 | 29.537 | Lengthening | 0.676666667 | 0.41 | 0.26666667 | 1.52E-20 | Shortening |
| *Fgfr1* | 55.948 | 76.795 | 20.847 | Shortening | 0.66 | 0.413333333 | 0.24666667 | 3.21E-18 | Shortening |
| *Arhgap1* | 18.654 | 39.379 | 20.725 | Lengthening | 0.906666667 | 0.686666667 | 0.22 | 2.82E-16 | Shortening |
| *Pa2g4* | 58.964 | 83.094 | 24.13 | Shortening | 1.0 | 0.663333333 | 0.33666667 | 1.75E-15 | Shortening |
| *Tmem43* | 31.347 | 60.744 | 29.397 | Shortening | 0.963333333 | 0.753333333 | 0.21 | 1.52E-12 | Shortening |
| *Ube2j2* | 4.755 | 49.213 | 44.458 | Shortening | 0.903333333 | 0.69 | 0.21333333 | 1.78E-05 | Shortening |
| *Map4* | 18.817 | 52.789 | 33.972 | Shortening | 0.62 | 0.373333333 | 0.24666667 | 1.98E-05 | Shortening |
| *Rabif* | 38.261 | 63.026 | 24.765 | Lengthening | 0.763333333 | 0.51 | 0.25333333 | 4.82E-04 | Shortening |
| ***Runx1*** | **44.367** | **74.673** | **30.306** | **Shortening** | **0.466666667** | **0.236666667** | **0.23** | **2.79E-03** | **Shortening** |
| *Kif18b* | 62.063 | 85.988 | 23.925 | Shortening | 0.463333333 | 0.153333333 | 0.31 | 8.25E-03 | Shortening |
| *Arsb* | 21.232 | 44.56 | 23.328 | Shortening | 0.86 | 0.646666667 | 0.21333333 | 0.008560812 | Shortening |
| *Aurka* | 18.894 | 64.202 | 45.308 | Shortening | 0.543333333 | 0.326666667 | 0.21666667 | 2.22E-02 | Shortening |
| *Isy1* | 28.497 | 50.0 | 21.503 | Shortening | 0.356666667 | 0.67 | -0.31333333 | 2.62E-02 | Lengthening |

**Table 1. Summary of alternative polyadenylation (APA) analysis results.**

Median PAU (polyadenylation usage) and mean PDUI (percentage distal usage index) values are shown for two conditions (H1 [SM22^Cre^ HX-SU], H2 [SM22*-Nudt21*^+/-^ HXSU]). diffPAU represents the difference in median PAU between conditions. UTR change indicates predicted transcript lengthening or shortening based on poly(A) site usage. PDUI_diff reflects the difference in distal usage index between H1 and H2, with lower PDUI values consistent with 3′UTR shortening. Adjusted P values were calculated using the Benjamini–Hochberg correction for multiple testing. Highlighted rows (e.g., Runx1) denote genes of particular interest for validation in the current study.

**Table S2**

| Sample ID | Age | Sex | Race | Diagnosis | mPAP (mmHg) |
| --- | --- | --- | --- | --- | --- |
| 1032 | 60 | F | C | Discarded Donor | N/A |
| 1033* | 26 | M | C | Discarded Donor | N/A |
| 1039* | 29 | M | C | Discarded Donor | N/A |
| 1040* | 43 | F | N/A | Discarded Donor | N/A |
| 1041* | 56 | M | H | Discarded Donor | N/A |
| 1042* | 28 | F | H | Discarded Donor | N/A |
| 1044 | 43 | F | C | Discarded Donor | N/A |
| 1046 | 52 | F | B | Discarded Donor | N/A |
| 1050 | 59 | M | H | Discarded Donor | N/A |
| 1053 | 30 | M | B | Discarded Donor | N/A |
| 1063 | 47 | F | C | Discarded Donor | N/A |
| 1067 | 73 | F | C | Discarded Donor | N/A |
| 1076 | 52 | F | H | Discarded Donor | N/A |
| 1072 | 62 | F | C | Discarded Donor | N/A |
| 1083 | 45 | F | B | Discarded Donor | N/A |
| 1098 | 28 | F | C | Discarded Donor | N/A |
| 1101 | 44 | F | B | Discarded Donor | N/A |
| 1105 | 53 | F | C | Discarded Donor | N/A |
|  |  |  |  |  |  |
| 132 | 49 | M | B | SSc-PAH | 33 |
| 148 | 58 | F | B | SSc-PAH | 42 |
| 177 | 49 | F | C | SSc-PAH | 52 |
| H20L0022 | 47 | F | H | SSc-PAH |  |
|  |  |  |  |  |  |
| 115 | 60 | F | B | SSc-ILD-PH | 28 |
| 157 | 41 | F | B | SSc-ILD-PH | 55 |
| 165 | 57 | F | C | SSc-ILD-PH | 46 |
| 168 | 52 | F | B | SSc-ILD-PH | 65 |
| 170 | 57 | M | B | SSc-ILD-PH | 27 |
| 190 | 69 | F | B | SSc-ILD-PH | 36 |
| H20L0023 | 69 | F | C | SSc-ILD-PH |  |
|  |  |  |  |  |  |
| 125 | 52 | F | C | PAH | 44 |
| 139 | 21 | F | N/A | PAH | 70 |
| 142 | 61 | F | N/A | PAH | 51 |
| 145 | 44 | F | C | PAH | 63 |
| 154 | 21 | F | C | PAH | 55 |
| 186 | 21 | F | C | PAH | 57 |
| 206 | 43 | M | H | PAH | 100 |
| 208 | 53 | F | C | PAH | 52 |
| H51 | 42 | F | N/A | PAH | N/A |

*****Denotes control samples which were used as controls for PAH comparisons. F: Female sex; M: Male sex; B: Black, C: Caucasian, H: Hispanic.

**Table S3: Mouse and Human primers used in study**

| Mouse RT-qPCR Primers |  |
| --- | --- |
| *Ptger3 F* | CCGGAGCACTCTGCTGAAG |
| *Ptger3 R* | CCCCACTAAGTCGGTGAGC |
| *Tbp F* | ATGATGCCTTACGGCACAGG |
| *Tbp R* | GTTGCTGAGATGTTGATTGCTG |
| *18s F* | GTAACCCGTTGAACCCCATT |
| *18s R* | GTAACCCGTTGAACCCCATT |
| *Runx1 F* | CTGCCCATCGCTTTCAAGGT |
| *Runx1 R* | GCCGAGTAGTTTTCATCATTGCC |
| *Cbfb F* | ACAAACACCTAGCCGGGAATA |
| *Cbfb R* | GCTGTGAAACTCTCACCTCCATT |
|  |  |
| Human RT-qPCR Primers |  |
| *CBFB F* | AGAAGCAAGTTCGAGAACGAG |
| *CBFB R* | CCTGAAGCCCGTGTACTTAATCT |
| *TBP F* | CCACTCACAGACTCTCACAAC |
| *TBP R* | CTGCGGTACAATCCCAGAACT |
| *18s F* | GTAACCCGTTGAACCCCATT |
| *18s R* | GTAACCCGTTGAACCCCATT |
| *PTGER3 F* | CGCCTCAACCACTCCTACAC |
| *PTGER3 R* | GACACCGATCCGCAATCCTC |
| *RUNX1 F* | ACCACTCCACTGCCTTTAAC |
| *RUNX1 R* | ACCACTCCACTGCCTTTAAC |
| *GAPDH F* | AAGGTGAAGGTCGGAGTCAAC |
| *GAPDH R* | GGGGTCATTGATGGCAACAATA |
|  |  |
| APA Primers Mouse |  |
| *Ptger3 total F* | CCGGAGCACTCTGCTGAAG |
| *Ptger3 total R* | CCCCACTAAGTCGGTGAGC |
| *Ptger3 distal F* | TGCAACGTACTAGACGCCAG |
| *Ptger3 distal R* | TCTGCCCATGACACTCAACC |
| *Runx1 total F* | CTGCCCATCGCTTTCAAGGT |
| *Runx1 total R* | GCCGAGTAGTTTTCATCATTGCC |
| *Runx1 distal F* | CCTCTATAGGGGTGGGGACA |
| *Runx1 distal R* | CCATTGAATCGGCTCCCTCC |
| *Cbfb total F* | ACAAACACCTAGCCGGGAATA |
| *Cbfb total R* | GCTGTGAAACTCTCACCTCCATT |
| *Cbfb distal F* | CCGGGCAAAACATTCACAGA |
| *Cbfb distal R* | CGACCACGGAAAGTATTGTAAGC |
|  |  |
| APA Primers Human |  |
| CBFB Total F | AGAAGCAAGTTCGAGAACGAG |
| CBFB Total R | CCTGAAGCCCGTGTACTTAATCT |
| CBFB Distal F | CATCTGTGCAGAGGATGCATTT |
| CBFB Distal R | AGCACTGTTTTCCACTCTGTATCT |
| PTGER3 Total F | CGCCTCAACCACTCCTACAC |
| PTGER3 Total R | GACACCGATCCGCAATCCTC |
| PTGER3 Distal F | TGTGGGCTTAGCATGGAACT |
| PTGER3 Distal R | ATGCCCTTAGCTGCATCACT |
| RUNX1 Total F | ACCACTCCACTGCCTTTAAC |
| RUNX1 Total R | ACCACTCCACTGCCTTTAAC |
| RUNX1 Distal F | GCCAGGTCAGTATTGATG |
| RUNX1 Distal R | GTCCTTAGAAACACACAC |
|  |  |
| mIR PCR Assays |  |
| miR-95 |  |
| miR-203a |  |
| miR-3163 |  |
| SMOR48 |  |

**Table S4. Antibodies utilized for research.**

| Target | Antibody |  | Concentration |
| --- | --- | --- | --- |
| aSMA | Sigma A5228 |  | IHC 1:1000 |
| Ki67 | abcam ab15580 |  | IHC 0.5 ug/mL |
| GAPDH | Cell Signaling Technology 2118s |  | WB 1:2000 |
| PTGER3 | Cayman Chemical 101760 |  | IHC 1:120,  WB 1:200 |
| RUNX1 | Proteintech 25315 |  | IHC 1:500,  WB 1:1000 |
| CPSF5 (NUDT21) | Proteintech 10322-1-AP |  | WB 1:1000 |
| CBFB | Abcam ab133600 |  | IHC 1:500,  WB 1:1000 |

**
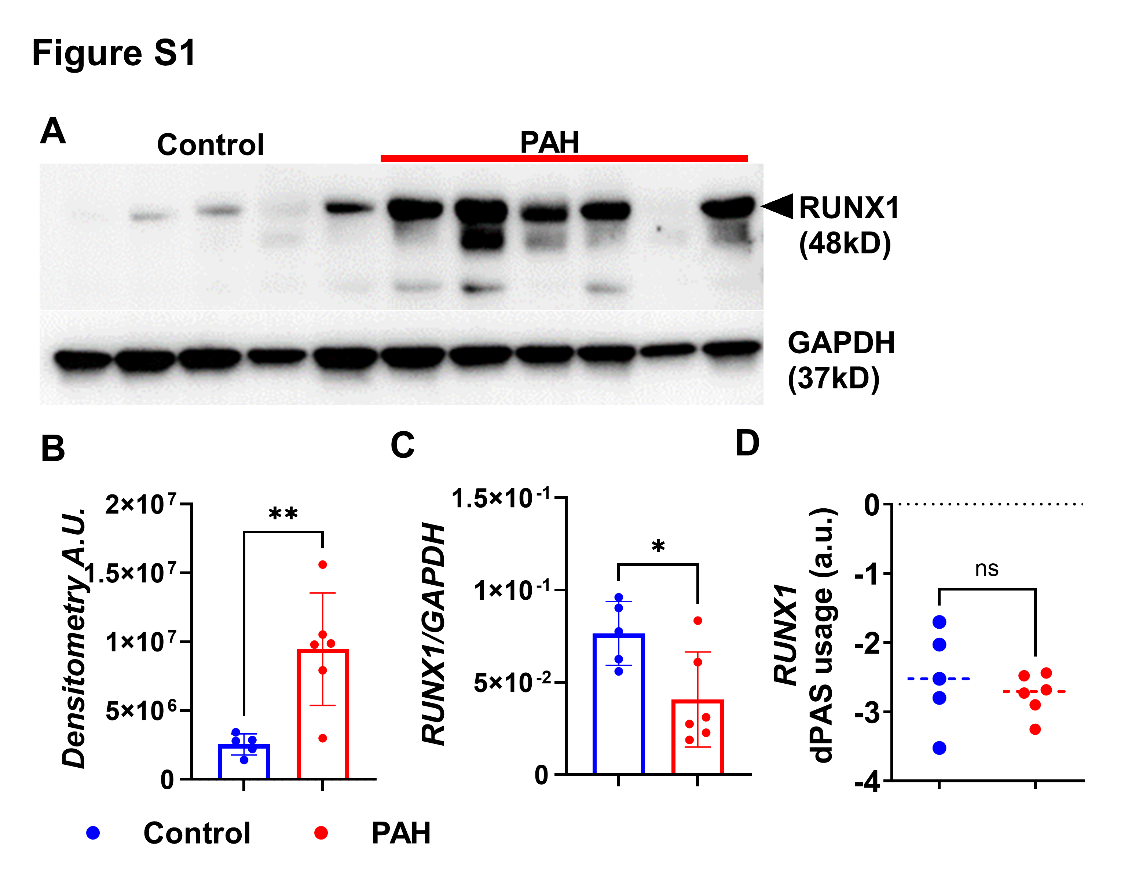
**

**Figure S1. RUNX1 is elevated in remodeled vessels from patients with PAH.**

(**A**) Western blot for RUNX1 (top band, denoted by arrowhead) and GAPDH (bottom bad) and corresponding densitometry for RUNX1 (**B**) from control or PAH isolated pulmonary arteries. **C**)*RUNX1* gene expression and (**D**) dPAS usage ratio for *RUNX1* from isolated pulmonary arteries. Significance level *P<0.05 represent Mann-Whitney comparisons between the control and PAH. Significance levels **P 0.001 to 0.01 and *P<0.05 represent Mann-Whitney comparisons between control and PAH. Biological *N numbers are as follows: Panel A: Control (N=5), PAH (N=7). Panels C and D: Control (N=5), PAH (N=6).*

**
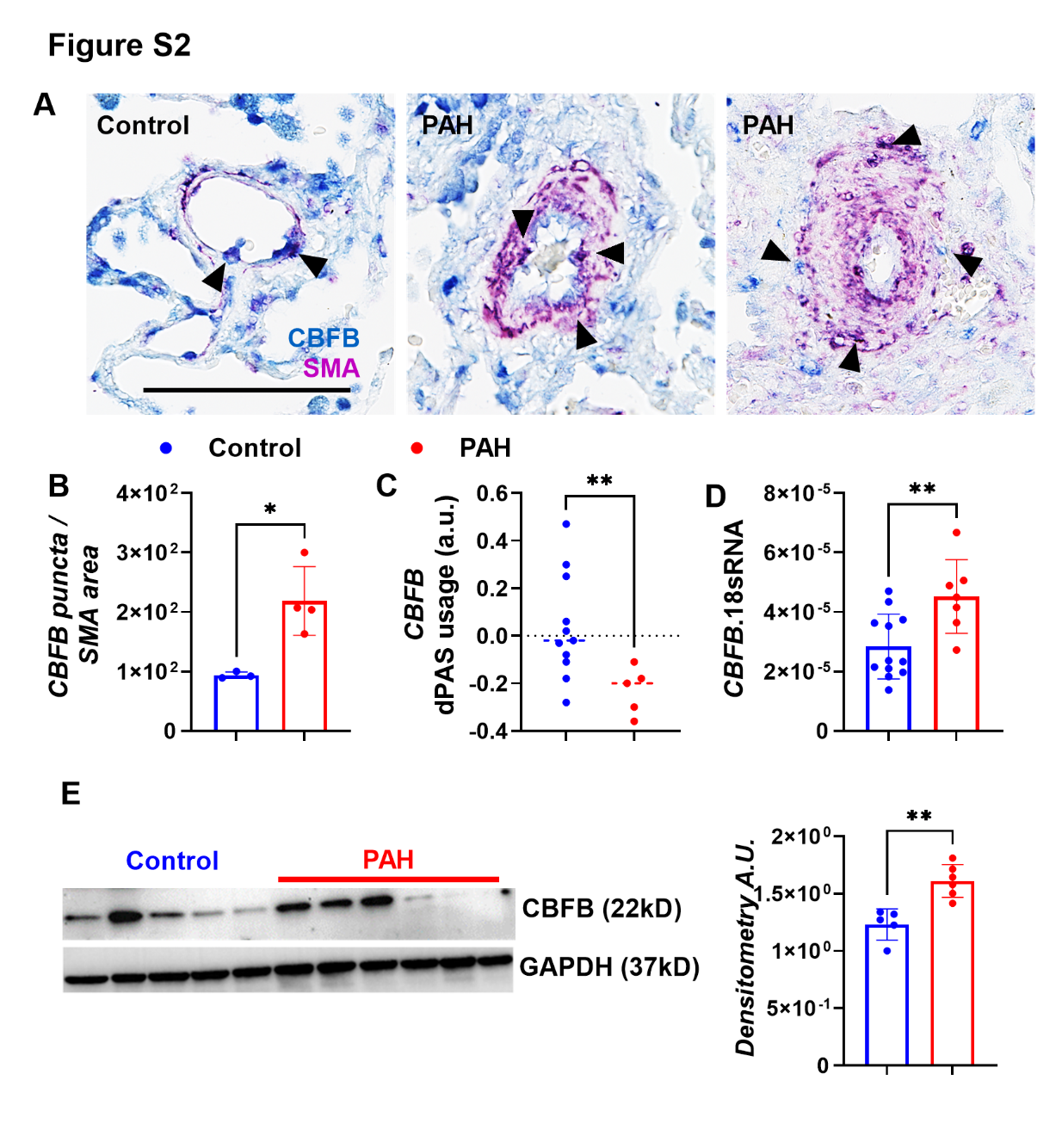
**

**Figure S2. *CBFB* undergoes 3’UTR shortening and increased expression in PAH.**

(**A**) Representative dual immunohistochemistry for vessels from control (left panel), two PAH patients (center and right panel), for SMA (pink/magenta signals) and CBFB (blue signals) and corresponding morphometric quantification for CBFB (**B**) The scale bar represents 100 µm and arrowheads point at CBFB cells within the remodeled vessels. (**C**) dPAS usage ratio for *CBFB* and (**D**) *CBFB* gene expression from isolated pulmonary arteries from control (blue) or PAH groups (red). (**E**) Western blot and corresponding densitometry for CBFB (top band) and GAPDH (bottom band) from control or PAH isolated pulmonary arteries. Significance levels **P 0.001 to 0.01 and *P<0.05 represent Mann-Whitney comparisons between the control and PAH. Biological *N numbers are as follows: Panel A and Panel C: Control (N=11), PAH (N=5). Panel D: Control (N=11), PAH (N=7). Panel E: Control (N=5), PAH (N=6.*

**
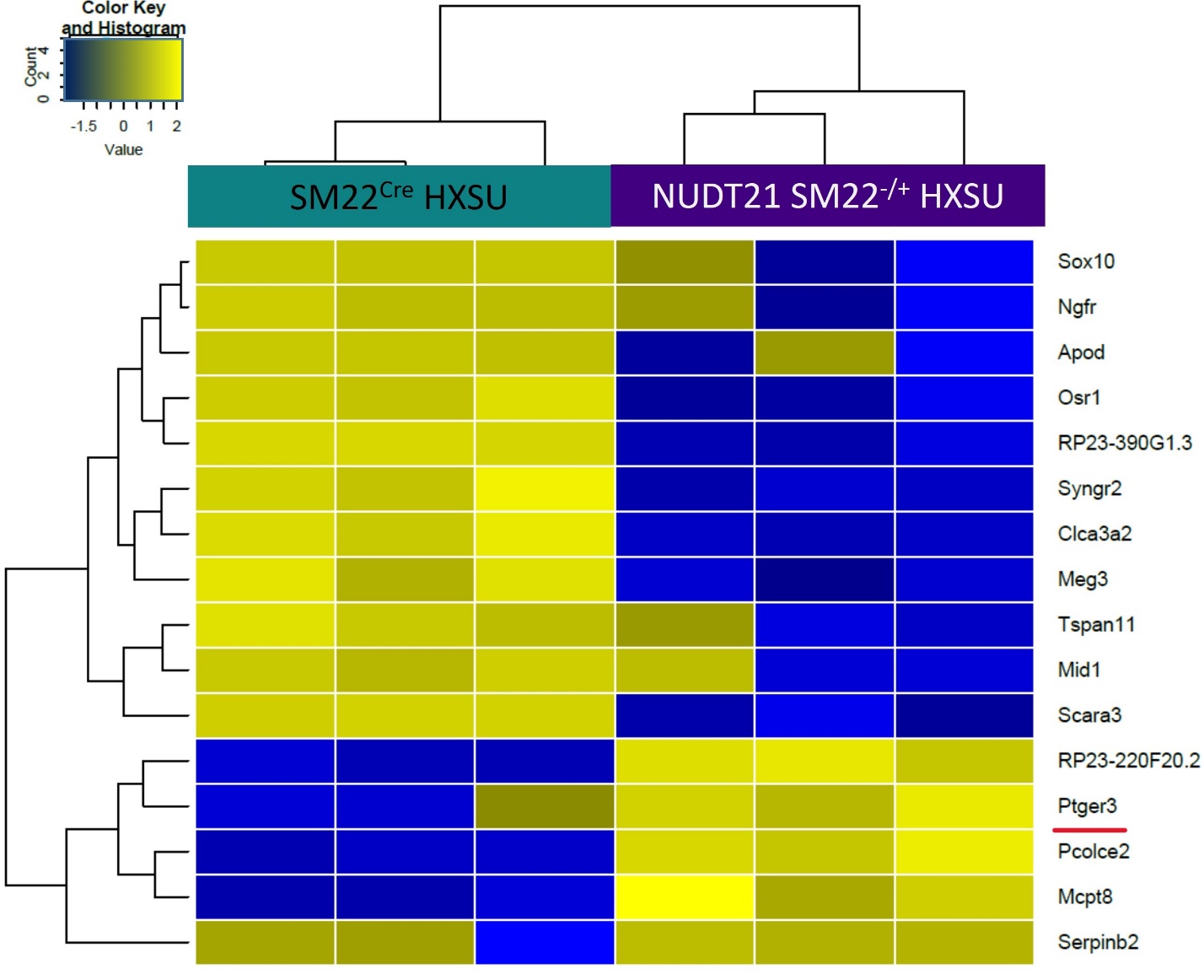
**

**Figure S3. Heat map identifying altered gene expression following hypoxia-sugen exposure in mice with reduced smooth muscle *Nudt21 expression*.** Heat map identifying differential gene expression determined through RNA-seq analysis from lung samples isolated from HX-SU exposed SM22^Cre^ (N=3) or SM22-NUDT21^-/+^ (N=3) exposed mice color key histogram denotes upregulated genes in yellow and downregulated genes in blue. Ptger3 is underlined in red.

**
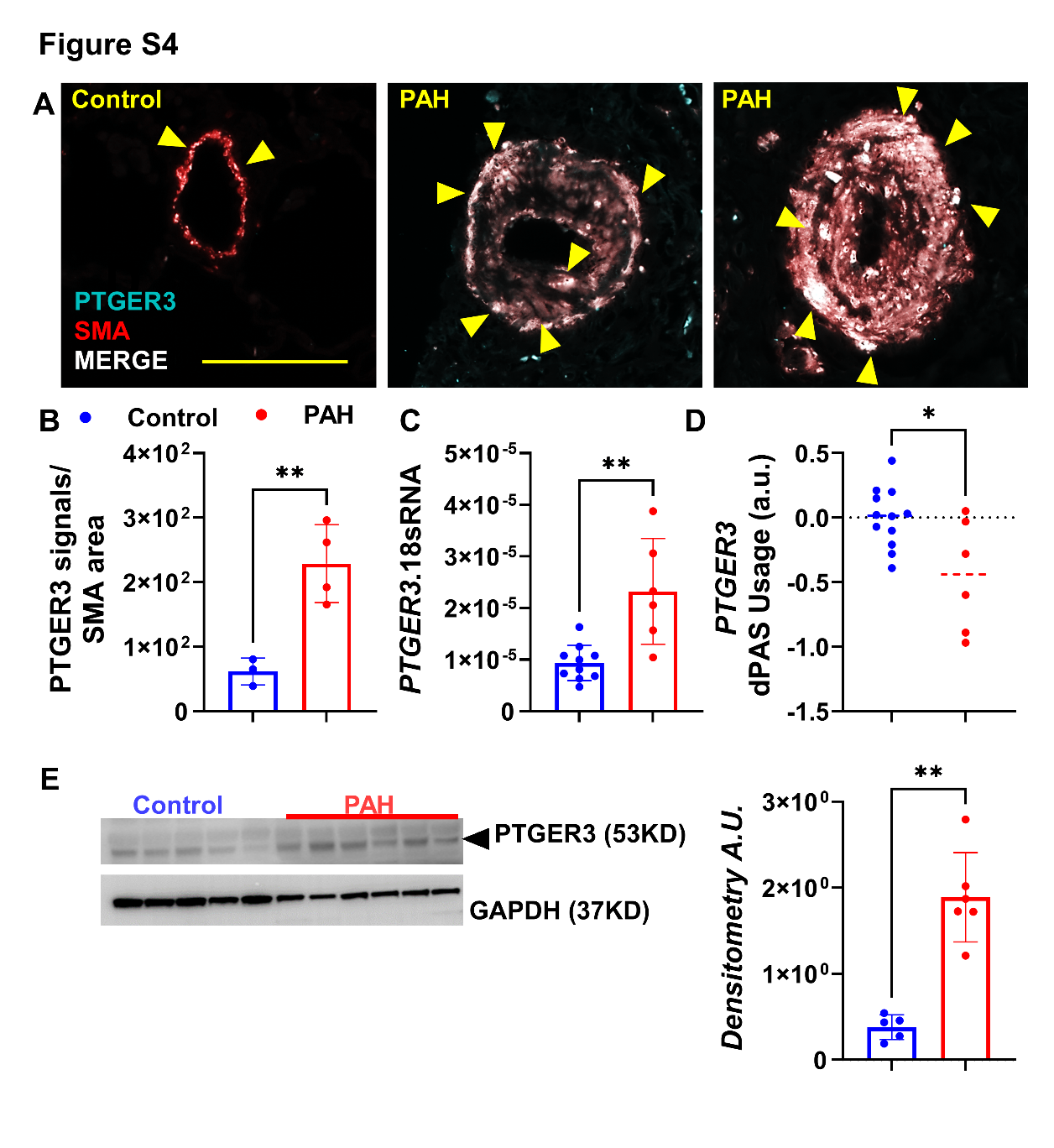
**

**Figure S4. *PTGER3* undergoes 3’UTR shortening and increased expression in PAH.**

**A**) Representative dual immunohistochemistry and corresponding morphometric analysis (**B**) for vessels from control (left panel), two PAH patients (center and right panel), for SMA (pink/magenta signals) and PTGER3 (cyan signals) The scale bar represents 100 µm and arrows point at CBFB cells within the remodeled vessels. *PTGER3* gene (**C**) expression and (**D**) dPAS usage ratio for *PTGER3* from isolated pulmonary arteries from control (blue) or PAH groups (red). (**E**) Western blot and corresponding densitometry for PTGER (lower band, denoted by arrowhead) and GAPDH (bottom band) from control or PAH isolated pulmonary arteries. Significance levels **P 0.001 to 0.01 and *P<0.05 represent Mann-Whitney comparisons between the control and PAH. Biological *N numbers are as follows: Panel B: Control (N=3), PAH (N=4); Panel C: Control (N=11), PAH (N=6); Panel D: Control (N=12), PAH (N=6) and Panel E: Control (N=5), PAH (N=6).*
